# The blistering warfare agent O-mustard (agent T) generates protein-adducts with human serum albumin useful for biomedical verification of exposure and forms intramolecular cross-links

**DOI:** 10.1007/s00216-024-05501-8

**Published:** 2024-08-31

**Authors:** Marc-Michael Blum, Wolfgang Schmeißer, Marina Dentzel, Horst Thiermann, Harald John

**Affiliations:** 1 Blum – Scientific Services, Björnsonweg 70d, 22587 Hamburg, Germany; 2 Bundeswehr Institute of Pharmacology and Toxicology, Neuherbergstraße 11, 80937 Munich, Germany

**Keywords:** Cross-links, Protein-adduct, Sulfur mustard, O-Mustard, Verification, Vesicant

## Abstract

The highly blistering sulfur mustard analogue agent T (bis(2-chloroethylthioethyl) ether), also known as O-mustard or oxy-mustard, is a common impurity in military grade sulfur mustard (SM) and a component of mixtures such as “HT” that are still found in old munitions. Together with sesquimustard (Q), it is the most important SM analogue and tightly regulated as a Schedule 1 chemical under the Chemical Weapons Convention. We report the adducts of T with nucleophilic Cys^34^ and other residues in human serum albumin (HSA) formed in vitro. A micro liquid chromatography electrospray ionization high-resolution tandem-mass spectrometry method (µLC-ESI MS/HR MS) was developed for the detection and identification of biomarker peptides alkylated by a T-derived hydroxyethylthioethyloxyethylthioethyl (*HETEOETE*)-moiety (as indicated by an asterisk below). Following proteolysis of T-exposed human plasma with pronase, the dipeptide Cys^34^*Pro and the single amino acid residue His* were produced. The use of proteinase K yielded Cys^34^*ProPhe and the use of pepsin generated ValThrGlu^48^*Phe, AlaGlu^230^*ValSerLysLeu, and LeuGlyMet^329^*Phe. Corresponding peptide-adducts of SM and Q were detected in a common workflow that in principle allowed the estimation of the mustard or mustard composition encountered during exposure. Novel adducts of Q at the Glu^230^ and Met^239^ residues were detected and are reported accordingly. Based on molecular dynamics simulations, we identified regular interactions of the Cys^34^(-*HETEOETE*)-moiety with several glutamic acid residues in HSA including Glu^86^, which is not an obvious interaction partner by visual inspection of the HSA crystal structure. The existence of this and other intramolecular cross-links was experimentally proven for the first time.

**Graphical abstract:**

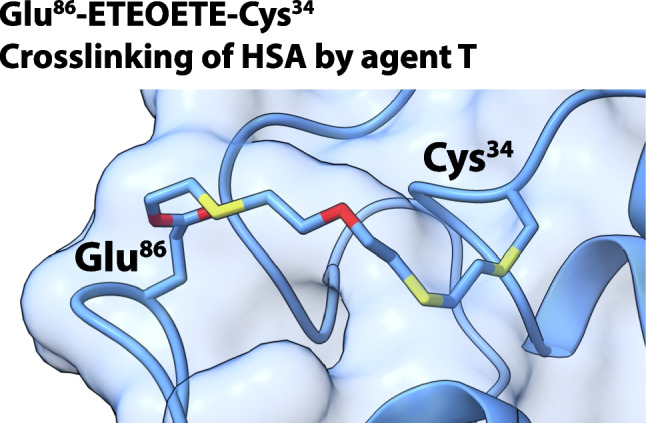

**Supplementary Information:**

The online version contains supplementary material available at 10.1007/s00216-024-05501-8.

## Introduction

**Fig. 1:**
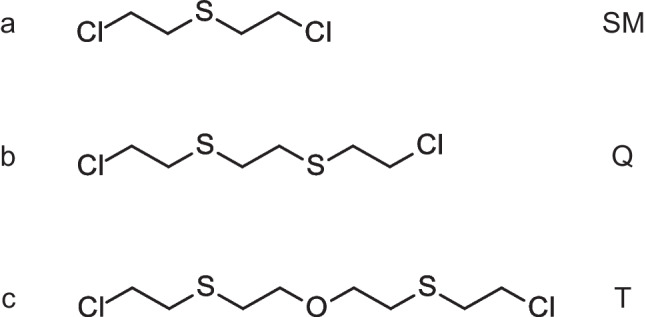
Chemical structures of alkylating sulfur mustard analogues: **a** sulfur mustard (SM), **b** sesquimustard (Q), **c** O-mustard; agent T (T)

Since the first use of bis(2-chloroethyl) sulfide, better known as sulfur mustard (SM, CAS 505–60-2, Fig. 1a), during World War I in the year 1917, blistering agents have been in the focus of defensive research. Research includes the detection, protection, decontamination, and medical countermeasures against these agents, that are also known as vesicants [1, 2]. It is still relevant today as the latest use of SM was reported in 2015 and 2016 by the so-called Islamic State (ISIS/ISIL) in Syria and Northern Iraq [3, 4]. SM not only causes painful blisters and erythema but is also characterized by prolonged wound healing requiring long-lasting medical attention of affected individuals [5–9]. It is cytotoxic and due to its reactivity with DNA it is mutagenic and classified as carcinogenic in humans [10, 11]. SM and structural analogues of higher toxicity like sesquimustard (Q, Fig. 1b) are listed in Schedule 1.A.04 of the annex on chemicals of the Chemical Weapons Convention (CWC) banning the production, stockpiling, and use of these agents apart from some very narrowly defined non-prohibited purposes such as defensive research [12]. Efficient implementation of the CWC requires the ability to detect chemical agents, their precursors, degradation, and biotransformation products in environmental and biomedical samples. For the analysis of biomedical samples, the Organisation for the Prohibition of Chemical Weapons (OPCW), the CWC’s implementing body, designates laboratories through successful participation in biomedical proficiency tests (BioPTs) [13].

We have recently reported on the formation of adducts of Q (Fig. 1b) with human serum albumin (HSA) for biomedical verification of exposure [14] complementing existing methods for the detection of exposure to SM [15–18]. Hemme et al. reported on similar adducts for other relevant bis(2-chloroethylthio)alkanes [19].

Another highly important sulfur-containing mustard analogue, also scheduled in 1.A.04, is bis(2-chloroethylthioethyl) ether (CAS 63918–89-8, Fig. 1c), also known as O-mustard, oxy-mustard, agent T, or O-lost, which is referred to herein simply as T. It is 3.5-times more blistering than SM and just like Q, it is a common impurity of SM and can serve as a forensic chemical signature [20]. In addition, it was part of “HT,” a tactical mixture of 60% w/w SM and 40% w/w T (and some other homologues) produced by the UK and the USA starting in the mid 1930s [21]. Similar mixtures employing T were produced and stockpiled in Germany during World War II as mixture “OB” with T contents as high as 70% w/w [22].

Due to its presence in old munitions, accidental exposure to T is not only a theoretical possibility. Due to the relatively low technological barrier of producing SM and other sulfur-containing mustard agents, their use in attacks on military personnel and civilian populations remains a credible risk [3, 4, 7–9].

Analysis of T, its hydrolysis, and oxidation products from environmental samples by means of gas (GC) and liquid chromatography (LC) coupled to mass spectrometry (MS) has been demonstrated [23–25]. Biotransformation products of T in human urine and their analysis employing LC-tandem MS (MS/MS) were described by Ash et al. [26]. However, products found in urine are cleared from the body within a few days after exposure.

In this work, we report on the adducts of T formed upon reaction with diverse amino acid residues in HSA. In analogy to the S-linked hydroxyethylthioethyl-adduct (*HETE*–HSA) found with SM [9, 15–18, 27] and the hydroxyethylthioethylthioethyl-adduct (*HETETE*-HSA) formed with Q [14], we refer to the adduct formed by T as hydroxyethylthioethyloxyethylthioethyl (*HETEOETE*-HSA). We have also employed molecular dynamics (MD) simulations to probe the structural flexibility of the protein for intramolecular linkages by the *ETEOETE linker* and confirmed these results experimentally.

## Materials and methods

### Chemicals and reagents

Acetone (p.A.), acetonitrile (ACN, gradient grade), dichloromethane (CH_2_Cl_2_, for GC), iso-propanol (iPrOH, p.A.), water (LiChrosolv), and proteinase K (recombinant from *Pichia pastoris*, PCR grade) were from Merck (Darmstadt, Germany); globulin-free HSA (> 99%) and pepsin from gastric mucosa from Sigma-Aldrich (Steinheim, Germany); formic acid (FA ≥ 98%) and NaOCl solution for decontamination (12% Cl_2_) from Carl Roth (Karlsruhe, Germany); pronase from *Streptomyces griseus* from Roche (lot no. 70327222, Mannheim, Germany); ammonium bicarbonate (NH_4_HCO_3_, ultra-grade, ≥ 99.5%) from Fluka (Buchs, Switzerland); and triple deuterated atropine (Atr-*d*_*3*_) from CDN Isotopes (Pointe Claire, Quebec, Canada). Pooled human ethylenediaminetetraacetic acid (EDTA) plasma was from Dunn Labortechnik (Asbach, Germany) and human EDTA plasma from different individuals was purchased from in.vent Diagnostica (Hennigsdorf, Germany).

The T hydrolysis product bis(2-hydroxyethylthioethyl) ether was purchased from BOC Sciences (Shirley, NY, USA). The diol was chlorinated to yield T in pure form as an oily liquid. Molecular structure and purity (> 99%) were confirmed by ^1^H- and ^13^C-nuclear magnetic resonance (NMR) spectroscopy:

bis(2-chloroethylthioethyl) ether: ^1^H-NMR (CDCl_3_, 400 MHz): δ 2.76 (t, 4H, *J* = 6.42 Hz), 2.92 (t, 4H, *J* = 6.42 Hz), 3.64 (t, 4H, *J* = 6.61 Hz), 3.65 (t, 4H, *J* = 6.61 Hz). C{H}-NMR (CDCl_3_, 100 MHz): δ 32.02, 34.80, 43.15, 71.06.

SM and Q were made available by the German Ministry of Defence and tested for integrity and purity (99%) in-house by NMR spectroscopy. Stock solutions of T and Q (150 mM each) were prepared in CH_2_Cl_2_ and that of SM in ACN, all further diluted with iPrOH to yield working solutions of diverse concentrations.

### Safety considerations

SM, Q, and T are potent vesicants and should only be handled by experienced and trained personnel using appropriate protective equipment in a properly working fume hood also requiring strict decontamination of all materials the poisons had contact with [14].

### Incubation of plasma and neat HSA with T

Plasma (1485 µL) was mixed with a working solution of T (15 µL, 6 mM in iPrOH/CH_2_Cl_2_ 96:4 v/v) resulting in 60 µM (reference) followed by a 2-h incubation at 37 °C and overnight at room temperature (RT) under gentle shaking. Standards were made with the following final T concentrations: 50 µM, 20 µM, 4 µM, 0.8 µM, 0.4 µM, 0.16 µM, and 0.08 µM. Blank plasma was produced accordingly, using a mixture of iPrOH/CH_2_Cl_2_ (96:4 v/v) instead of T working solution. Neat HSA (990 µL, 40 mg/mL in PBS) was mixed with T stock solution (10 µL) for incubation under the same conditions.

### Plasma sample preparation

Plasma references, standards, and blank (100 μL, each) were pipetted into ultrafiltration (UF) devices (Amicon Ultra-0.5 centrifugal filter unit, 0.5 mL, molecular weight cut-off, MWCO, 10 kDa, Merck Millipore, Billerica, MA, USA) and mixed for proteolysis with either (i) pepsin solution (100 µL, 2 mg/mL in 10% v/v FA) and FA (50 µL, 10% v/v, incubation for 4 h at 37 °C) or with (ii) pronase solution (100 µL, 60 mg/mL in 50 mM NH_4_HCO_3_) and NH_4_HCO_3_ buffer (300 µL, 50 mM, incubation for 2 h at 37 °C) or with (iii) proteinase K solution (100 µL, 20 mg/mL in 50 mM NH_4_HCO_3_) and NH_4_HCO_3_ buffer (300 µL, 50 mM, incubation for 2 h at 47 °C). Afterwards, mixtures were subjected to UF (12,270 RCF, 10 min, 15 °C) yielding biomarker peptide containing filtrates. When using pepsin, two additional UF steps were carried out, each after adding FA (50 µL, 5% v/v) to the retentate. Filtrates obtained from the different proteolysis steps (60 µL, each) were mixed with Atr-*d*_*3*_ solution (120 µL, each, 3 ng/mL in 0.5% v/v FA) prior to micro liquid chromatography-electrospray ionization high-resolution tandem-mass spectrometry (µLC-ESI MS/HR MS) using either a hybrid quadrupole time-of-flight instrument (TT5600^+^) or an Orbitrap mass spectrometer (Orbitrap).

### µLC-ESI MS/HR MS (TT5600^+^) analysis for biomarker detection

The chromatographic system consists of a microLC 200 pump (Eksigent Technologies LLC, Dublin, CA, USA) and a HTC xt DLW autosampler (CTC Analytics, Zwingen, Switzerland) with a 20-μL sample loop (Sunchrom, Friedrichsdorf, Germany) and was controlled by the Eksigent control 4.2 software (ABSciex, Darmstadt, Germany).

Ultrafiltrates finally obtained after the diverse sample preparations were separated on an Acquity HSS T3 column (50 × 1.0 mm I.D., 1.8 μm, 100 Å, Waters, Eschborn, Germany) protected by a precolumn (Security Guard™ Ultra Cartridges UHPLC C18 peptide 2.1 mm I.D., Phenomenex, Aschaffenburg, Germany) carried out in gradient mode of solvent A (0.05% v/v FA) and solvent B (ACN/H_2_O 80:20 v/v, 0.05% v/v FA) at 60 °C with 30 µL/min after a 5-min equilibration period under starting conditions: t [min]/B [%]: 0/2; 11.0/50; 11.5/95; 13.5/95; 14.0/2; 15.0/2.

For mass spectrometric detection in product ion scan (PIS) mode after collision-induced dissociation (CID), a high-resolution hybrid mass spectrometer TripleTOF 5600^+^ (TT5600^+^, ABSciex) was used operating with positive ESI monitoring ions in the range from *m*/*z* 50 to *m*/*z* 900. Masses of precursor ions and qualifier ions (Qual I–Qual III) of biomarkers are listed in Table 1. The PeakView 2.1 and MultiQuant 2.1.1 software (ABSciex) were used for data interpretation. Extracted ion chromatograms (XICs) of product ions isolated with unit resolution of the quadrupole were deduced from the total ion chromatograms (TICs) with a tolerance of ± 0.005 Th. The following MS settings were used for simultaneous PIS analysis: ion spray voltage floating (ISVF) 4.5 kV; declustering potential (DP) 60 V; curtain gas (CUR) 2.07 × 10^5^ Pa (30 psi); heater gas (GS1) 2.76· × 10^5^ Pa (40 psi); turbo ion spray gas (GS2) 3.45 × ·10^5 Pa^ (50 psi); temperature (TEM) 200 °C; collision energy spread (CES) 3 V; ion release delay (IRD) 67 ms; ion release width (IRW) 25 ms; and accumulation time 50 ms.

**Table 1:** Mass spectrometric parameters and characteristics for detection of biomarker adducts with T

Compound	Precursor ion	*m*/*z*	Qual I [*m*/*z*] (CE [V])	Qual II [*m*/*z*] (CE [V])	Qual III [*m*/*z*] (CE [V])	*t*_R_ [min]	LOI [µM]	Qualifier ion ratio for LOI
C^34^(-*HETEOETE*)P ^a^	[M + H]^+^	427.139	181.034 (32)	217.065 (32)	105.037 (29)	6.5	0.08	III/I (32.0%)
C^34^(-*HETEOETE*)PF ^b^	[M + H]^+^	574.207	181.034 (42)	209.067 (28)	364.133 (28)	9.2	0.08	II/I (29.6%)
AE^230^(-*HETEOETE*)VSKL ^c^	[M + 2H]^2+^	427.722	181.035 (22)	105.037 (26)	750.405 (18)	6.4	4.0	III/I (70.6%)
LGM^329^(-*HETEOETE*)F ^c^	[M + H]^2+^	338.149	181.035 (16)	209.065 (12)	467.232 (12)	6.0	0.4	II/I (57.7%)
VTE^48^(-*HETEOETE*)F ^c^	[M + H]^+^	703.304	181.035 (45)	209.065 (39)	137.009 (56)	8.9	4.0	II/I (31.9%)
H(-*HETEOETE*) ^a^	[M + H]^+^	364.136	181.034 (28)	137.009 (36)	209.066 (24)	3.4 // 4.0 ^d^	0.16	II/I (15.0%)
Atr-d_3_	[M + H]^+^	293.194	127.131 (42)	93.070 (42)	-	7.1	n.d	II/I (49.1%)

*CE*, collision energy; *LOI*, limit of identification; *Qual*, qualifier ion; *t*_*R*_, retention time

^a^Obtained after proteolysis with pronase; ^b^obtained after proteolysis with proteinase K; ^c^obtained after proteolysis with pepsin; ^d^two isobaric adducts of His (H) were obtained bearing the alkylation either at N^1^ od N^3^ of the amino acid side chain exhibiting different *t*_R_. According to the order of elution reported by Hemme et al. [19], the peak at *t*_R_ 3.4 min corresponded to N^1^ and that at *t*_R_ 4.0 min to N^3^-adduction. The origin of the single alkylated His (amino acid number) cannot be specified

Due to reasons of clarity, the one-letter code for amino acids is used

Automatic mass calibration after every fourth run was performed by infusing a calibration solution (500 µL/min, using the calibrant delivery system, CDS, ABSciex) containing reserpine to monitor product ions in the range from *m*/*z* 174.091 to *m*/*z* 609.281. The MS system was controlled by the Analyst TF 1.7.1 software (ABSciex).

### µLC-ESI MS/HR MS (Orbitrap) for cross-link analysis

To clarify T-induced cross-links in HSA, incubation mixtures with neat HSA were prepared by proteolysis with proteinase K as well as pepsin followed by precipitation and analysis of the supernatant by µLC-ESI MS/HR MS (Orbitrap). Analyses were carried out by an initial HR full scan (full width at half-maximum, fwhm, 70,000) followed by parallel reaction monitoring (PRM, fwhm 17,500) targeting postulated cross-linked peptides containing Cys^34^ linked either to Glu^86^ or Glu^48^ or Glu^45^. Cross-links (Cys^34^-*ETEOETE*-Glu^86^, Cys^34^-*ETEOETE*-Glu^48^, Cys^34^-*ETEOETE*-Glu^45^) were deduced from MD simulations as described below and corresponding cross-linked peptides were postulated based on the known amino acid sequence of HSA (UniProtKB P02768, numbering used herein in general does not consider the pro and signal peptide).

The chromatographic system comprised a pump (MicroPro, Eldex Laboratories, Napa, CA, USA), a column oven (Mistral, Spark Holland, Emmen, The Netherlands), and an autosampler (Integrity, Spark Holland) that were controlled by the accompanying software MicroPro 1.0 (build 1.0.57, Eldex) and Integrity autosampler control (build 1.00.57). Samples (20 µL) were chromatographed at 40 °C using the same stationary and mobile phase as mentioned above with the following gradient at 30 µL/min: t [min]/B [%]: 0/2; 2.5/10; 3.0/20; 15.0/45; 16.0/98; 18.0/98; 19.0/2; 20.0/2 (equilibration for 5 min under starting conditions). The µLC system was connected with a QExactive plus Orbitrap mass spectrometer via the HESI II ion source (both Thermo Scientific, Bremen, Germany). The Xcalibur vers. 4.3.73.11 and Tune 2.11 QF1 software with FreeStyle 1.8 SP1 were used for system control and data interpretation (all Thermo Fisher Scientific). HR MS and MS/HR MS detection was based on the method described recently [28].

For initial full scan MS measurement ions were monitored in the mass range from *m*/*z* 100 to *m*/*z* 1500 with an automated gain control (AGC) of 3 × 10^6^ charges and a maximum injection time (IT) of 100 ms between 3.0 and 17.5 min. Additional settings were as follows: sheath gas flow 23 arbitrary units (a.u.), auxiliary gas flow 8 a.u., sweep gas flow 1 a.u., spray voltage 3.5 kV, capillary temperature 250 °C, S-lens RF level 50 a.u., and auxiliary gas heater temperature 125 °C. In the PRM mode, product ions were detected with first fixed mass at *m*/*z* 50 (AGC 2 × 10^5^ charges, maximum IT 50 ms, isolation window 2.0 m/*z*, isolation offset to 0.5 m/*z*, normalized collision energy, NCE, normalized to *m*/*z* 200, *z* = 1). Mass calibration was done with lock masses of protonated ubiquitous molecules (dioctyl phthalate C_24_H_39_O_4_, *m*/*z* 391.28429, and *n*-butyl benzenesulfonamide C_10_H_16_O_2_NS, *m*/*z* 214.08963).

### Selectivity

Plasma from six individuals was treated as blank subjected to the standard protocol for sample preparation followed by µLC-ESI MS/HR MS (TT5600^+^) analysis for all biomarker peptides (Table 1) derived from pepsin-, pronase-, and proteinase K–catalyzed cleavage to test for the presence of any interference.

### Linear range and lower limit of identification

Standards (*n* = 3) and blank were prepared with all three enzymes separately and analyzed by µLC-ESI MS/HR MS (TT5600^+^) as described above. Biomarker peak areas deduced from respective XICs of product ions (Table 1) were plotted against the concentration of T. The limit of identification (LOI) was defined as the lowest concentration of T in plasma at which ion ratios (peak area ratios) of at least two product ions of the biomarker were similar to those obtained from the respective reference in all three replicates.

### Stability of biomarkers in the autosampler

Prepared references were stored in the autosampler at 15 °C and frequently analyzed by µLC-ESI MS/HR MS (TT5600^+^) over a period of 24 h to monitor the relative concentration profiles of all biomarker peptides.

### Freeze-and-thaw cycles

Three aliquots (500 µL, each) of freshly prepared reference (60 µM T) were analyzed immediately after the incubation period described above (*t*_0_) and after one (*t*_1_), two (*t*_2_), and three (*t*_3_) freeze-and-thaw cycles by µLC-ESI MS/HR MS (TT5600^+^). Each cycle consists of a 24-h storage period at − 20 °C ending with thawing and 1-h storage at RT prior to analysis. Peak areas of biomarkers produced with pepsin, pronase, and proteinase K (Table 1) were determined to follow their relative concentrations.

### Co-incubation of mustard agents in plasma

Working solutions of mustard agents were mixed yielding a molar ratio of 10:1:1 (500 µM SM, 50 µM Q, and 50 µM T). This mixture (44 µL) was immediately added to plasma (1956 µL, yielding final concentrations of 11 µM SM, 1.1 µM Q, and 1.1 µM T) followed by incubation under conditions described above. Analysis by µLC-ESI MS/HR MS (TT5600^+^) was carried out according to the standard protocol using all three enzymes, separately. Blank plasma was handled accordingly without the presence of mustard agents. Adducts of Q and SM were simultaneously detected with those of T by µLC-ESI MS/HR MS (TT5600^+^) based on the methods described recently [14, 15]. Newly introduced biomarkers of Q AE^230^(*-HETETE*)VSKL and LGM^329^(*-HETETE*)F were analyzed as summarized in Table ESM 1 in the Electronic Supplementary Material (ESM) applying the chromatographic conditions described above.

### MD simulations

MD simulations were carried out employing the same force field and conditions as in our previous work on Q [14]. MD simulations were carried out using the GROMACS package [29] (v. 2020.2) employing the GROMOS96 53A7 force field [30]. The topology for Cys adducted by T (Cys^34^(-*HETEOETE*) was created using the Automated Topology Builder (ATB) version 3.0 [31] and resulting values (ATB Molecule ID 705026) were used to add the modified amino acid to the force field. Coordinates for HSA were obtained from the protein database (PDB entry: 1AO6) and used for the apo protein. The starting structure for the construction of the Cys^34^(-*HETEOETE*) variant of HSA was the same used for the construction of the corresponding Cys^34^(-*HETETE*) variant for Q [14] which resulted from a production simulation of the apo protein in which a simulation frame was selected where the Cys^34^ residue was solvent exposed and the adducted mustard agents side chains could be added without steric hindrance. The Cys^34^ residue of HSA was modified using UCSF ChimeraX [32] so that the *HETEOETE*-moiety points either directly into the solvent or seeks interactions with amino acid side chains in the groove between the two helices adjacent to Cys^34^. The two variants were centered in a cubic unit cell with a distance between solute and box edge of 1.0 nm that was subsequently filled with simple point charge (SPC) water [33]. Fourteen sodium ions were added to balance the charge. Steepest descent minimization was performed, followed by 200 ps of canonical (NVT) equilibration and 200 ps of equilibration under an isothermal-isobaric (NPT) ensemble. Production simulations of 100 ns for the two Cys^34^(-*HETEOETE*) variants followed. Positional restraints were applied to the protein during equilibration and released for the production run. All bond lengths were constrained using the LINCS method, allowing a 2-fs time step [34]. The Verlet cut-off scheme was applied [35]. Long-range electrostatic interactions were calculated with the particle mesh Ewald (PME) method [36, 37]. Simulations utilized the velocity-rescaling thermostat [38] and Parrinello-Rahman barostat [39, 40]. Further details can be found in the Electronic Supplementary Material (Protocol ESM 1).

## Results and discussion

### Biomarkers obtained from plasma

Earlier studies performed by our group focused on diverse peptide biomarkers derived from HSA-adducts with SM [9, 15, 16, 18, 27, 41, 42] and Q [14]. Following pronase-catalyzed proteolysis, adducts of the dipeptide Cys^34^Pro were found: Cys(*-HETE*)Pro [9, 15, 16, 27, 42] and Cys(*-HETETE*)Pro [14]. After use of proteinase K, the adducted tripeptide Cys^34^ProPhe was detected: Cys(*-HETE*)ProPhe [15, 17] and Cys(*-HETETE*)ProPhe [14]. When using pepsin, the adducted hexapeptide AlaGlu^230^(*-HETE*)ValSerLysLeu [15] and the tetrapeptide LeuGlyMet^329^(*-HETE*)Phe [41] were produced. Furthermore, Hemme et al. and John et al. reported on the adduction of diverse His [19, 43] and Glu residues [43] in serum albumin that are cleaved as single amino acid markers after pronase-catalyzed cleavage. Therefore, we assumed that related adducts of T should be generated accordingly containing the *HETEOETE*-moiety alkylating either Cys^34^, Glu^230^, Met^329^, or any His residue.

**Fig. 2:**
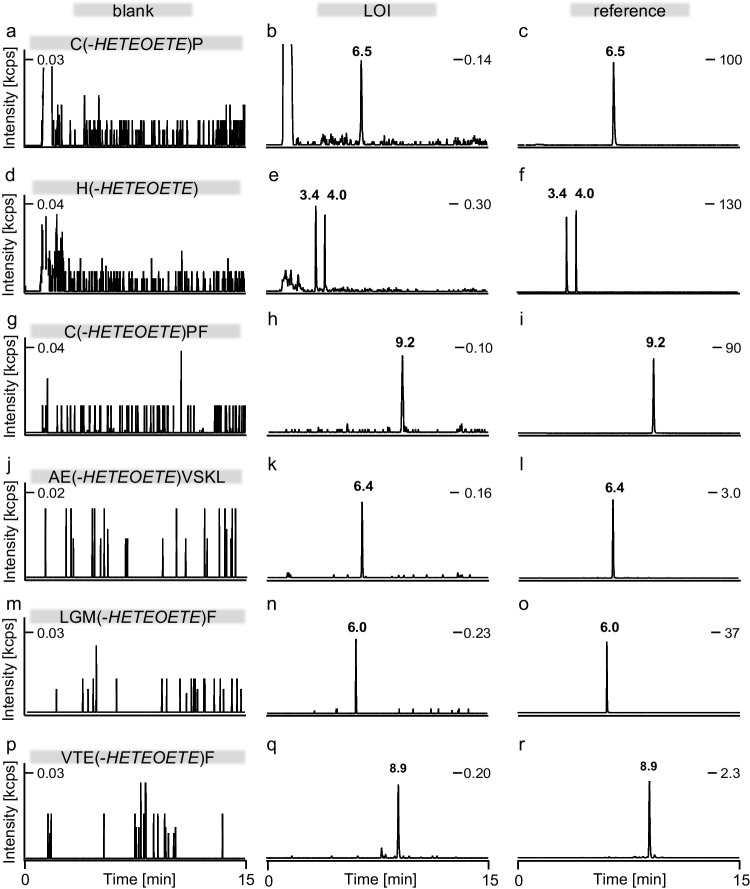
Representative extracted ion chromatograms of adduct biomarkers of T. References were obtained from plasma incubated with 60 µM T and analyzed by µLC-ESI MS/HR MS (TT5600 +). Plasma proteins were proteolyzed either with pronase yielding Cys(-*HETEOETE*)Pro, [M + H]^+^ m/z 427.139 (**a**–**c**) and His(-*HETEOETE*), [M + H]^+^ m/z 364.136 (**d**–**f**), or with proteinase K yielding Cys(-*HETEOETE*)ProPhe, [M + H]^+^ m/z 574.207 (**g**–**i**) or with pepsin producing AlaGlu(-*HETEOETE*)ValSerLysLeu, [M + 2H]^2+^ m/z 427.722 (**j**–**l**) and LeuGlyMet(-*HETEOETE*)Phe, [M + H]^2+^ m/z 338.149 (**m**–**o**) and ValThrGlu(-*HETEOETE*)Phe, [M + H]^+^ m/z 703.304 (**p**–**r**). For reasons of clarity, traces are assigned with the common one-letter code of amino acids and only the most intense common product ion of the product ion scans (m/z 181.035 ± 0.005) is depicted. *HETEOETE*, hydroxyethylthioethyloxyethylthioethyl; LOI, limit of identification

By applying a PIS method (Table 1) for the targeted analysis of protease-treated plasma using µLC-ESI MS/HR MS (TT5600^+^), we succeeded in the detection of the following markers: Cys(*-HETEOETE*)Pro (Fig. 2b, c), His(*-HETEOETE*) (Fig. 2e, f), Cys(*-HETEOETE*)ProPhe (Fig. 2h, i), AlaGlu(*-HETEOETE*)ValSerLysLeu (Fig. 2k, l), and LeuGlyMet(*-HETEOETE*)Phe (Fig. 2n, o). In addition, the alkylated tetrapeptide ValThrGlu^48^(-*HETEOETE*)Phe (Fig. 2q, r) was detected and identified as a novel peptide-adduct. Detection of this peptide-adduct was based on theoretical assumptions on cleavage products produced by pepsin. However, its peak intensity was much smaller than those of the other markers obtained from the plasma reference making the peptide less favorable for verification thus being excluded from validation.

**Fig. 3:**
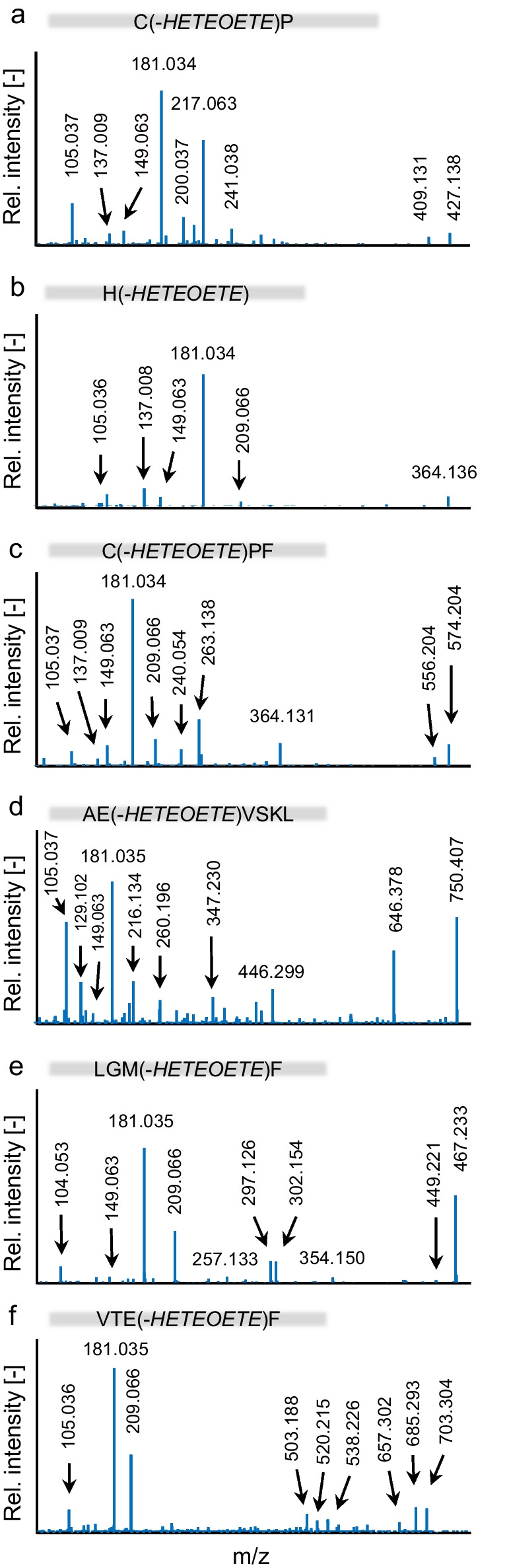
Product ion spectra of adduct biomarkers of T. Spectra were extracted from µLC-ESI MS/HR MS (TT5600 +) analysis in the product ion scan (PIS) mode of a plasma reference incubated with T (60 µM) as illustrated in Fig. 2. Precursor ions were at **a** m/z 427.1 for single protonated Cys(-*HETEOETE*)Pro (Table 2), **b** m/z 364.1 for single protonated His(-*HETEOETE*) (Table ESM 2), **c** m/z 574.2 for single protonated Cys(-*HETEOETE*)ProPhe (Table ESM 3), **d** m/z 427.7 for double protonated AlaGlu(-*HETEOETE*)ValSerLysLeu (Table ESM 4), **e** m/z 338.1 for double protonated LeuGlyMet(-*HETEOETE*)Phe (Table ESM 5) and **f** m/z 703,3 for the single protonated ValThrGlu(-*HETEOETE*)Phe (Table ESM 6). Tables referred to in parentheses provide the structural assignment of product ion signals labelled in the spectra. For reasons of clarity, mass spectra are assigned with the common one-letter code of amino acids

The product ion spectra of mentioned adducts are illustrated in Fig. 3. The structural assignment of product ions of Cys(-*HETEOETE*)Pro is listed in Table 2 and that of the others in Table ESM 2–Table ESM 6.

**Table 2:**
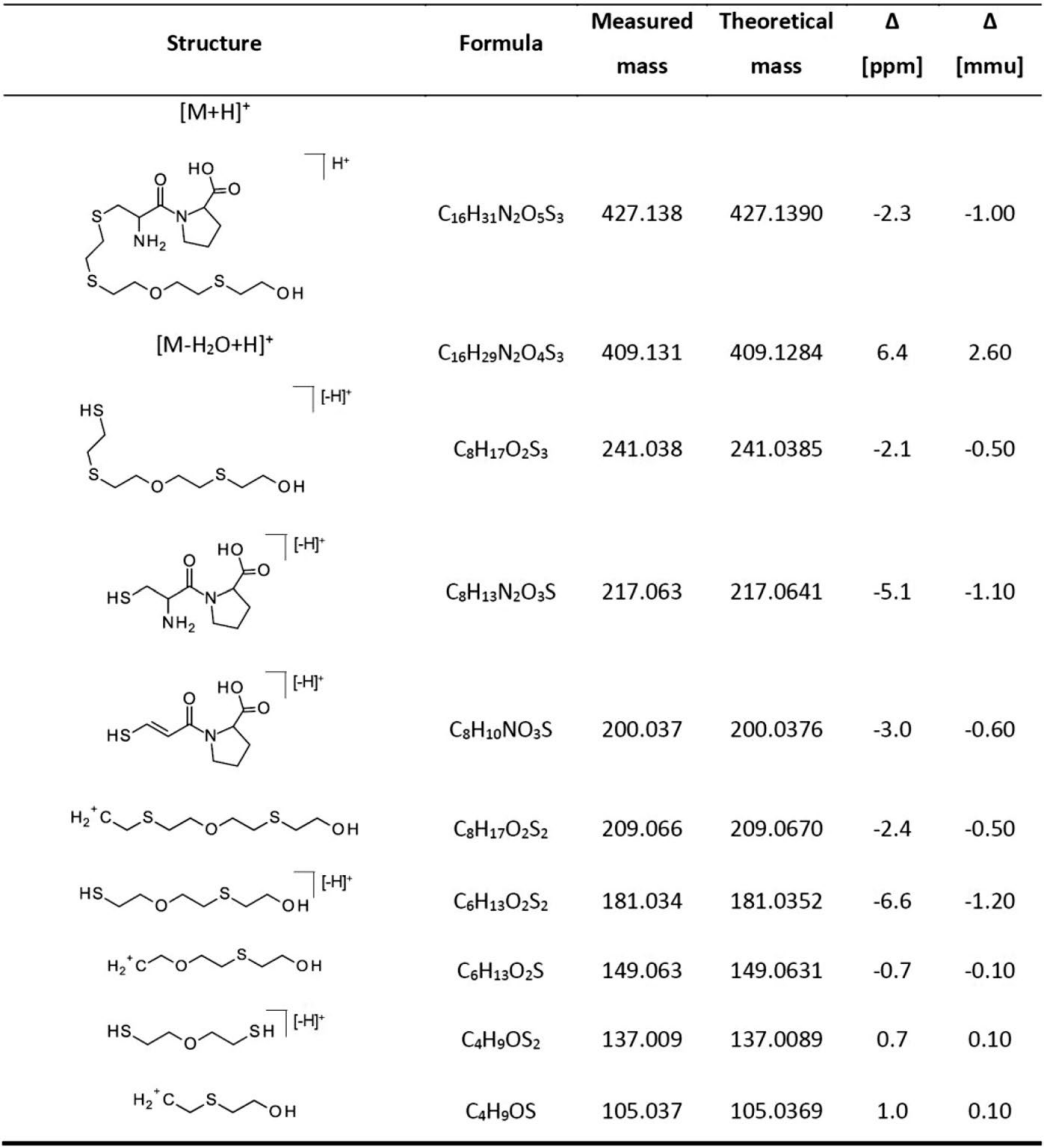
Product ions of single protonated Cys(-*HETEOETE*)Pro

The individual most intense product ions of these biomarkers used for their most selective and sensitive monitoring are listed in Table 1 presenting the qualifier ion I (Qual I) as the most intense followed by Qual II and Qual III. Within smallest mass deviations (< 7 ppm), every marker exhibited the same Qual I (*m*/*z* 181.035) that represents the [*HETEOET*]^+^ product ion (HO-C_2_H_4_-S-C_2_H_4_-O-C_2_H_4_-S^+^) derived from the attached alkyl-chain (Table 2, Fig. 3, Table ESM 2–Table ESM 6). Additional intense product ions derived from the alkyl-chain were detected at *m*/*z* 209.067 ([HO-C_2_H_4_-S-C_2_H_4_-O-C_2_H_4_-S-C_2_H_4_]^+^), at *m*/*z* 137.009 ([HO-C_2_H_4_-S-C_2_H_4_-S]^+^), and at *m*/*z* 105.037 ([HO-C_2_H_4_-S-C_2_H_4_]^+^) (Table 1, Fig. 3, Table ESM 2–Table ESM 6). These results confirmed that T reacts with HSA at diverse amino acids also known as targets for alkylation by SM [9, 15, 16, 41] and Q [14]. In addition, product ions mentioned above may serve as highly selective diagnostic ions for exposure to T. Accordingly, we characterized the suitability of these adducts as biomarkers as follows.

### Linear range and LOI

Based on Qual I (*m*/*z* 181.035), LOI values described as the concentration of T in plasma were found as listed in Table 1. Linear ranges for biomarkers were found between the LOI and 50 µM T representing the highest concentration tested. Chromatograms obtained from prepared plasma samples spiked at the LOI level are illustrated in the second panel of Fig. 2.

### Selectivity

No interference for any of the biomarkers for T were found in the blanks obtained from six individuals thus documenting high selectivity of the method very well suited to prove exposure to T. Representative blanks are illustrated in the first panel of Fig. 2.

### Stability of biomarkers in the autosampler

*HETEOETE*-adducts of Cys^34^Pro, Cys^34^ProPhe, AlaGlu^230^ValSerLysLeu, LeuGlyMet^329^Phe and His were stable for at least 24 h at 15 °C and did not show any trend of degradation. Peak areas of the respective markers only varied between 2 and 8% (results not shown). Therefore, these alkylated markers are very well suited even for analyzing larger sets of samples.

### Freeze-and-thaw cycles

After four freeze-and-thaw cycles, no trend of degradation was observed for any of the adducted amino acids (RSD of peak areas ≤ 11%, results not shown) thus documenting the suitability of plasma samples for storage and repeated analysis.

### Adduct detection after co-incubation

The mixture of mustard agents (SM:Q:T, 10:1:1) simulated military grade sulfur mustard resulting from long-term storage and side-reactions during synthesis. Following co-incubation of plasma with SM, Q, and T, respective adducts at Cys^34^, Glu^230^, and Met^329^ were detected simultaneously. The simultaneous traceability of all adducts allowed monitoring of the entire pattern of blister agents to characterize the poison mixture which might be of relevance for toxicological, forensic, and legal aspects. Adducts of Q at Glu^230^ and Met^329^ yielding the biomarker peptides AlaGlu(-*HETETE*)ValSerLysLeu and LeuGlyMet(-*HETETE*)Phe are reported herein also for the first time. Corresponding MS/HR MS spectra are shown in Figure ESM 1 and structural assignments of product ions are listed in Table ESM 7 and Table ESM 8.

### Analysis of HSA Cys^34^(-*HETEOETE*) variants using MD simulations—potential for intramolecular cross-linking by T

As the most abundant carrier protein in blood, HSA has been in the focus of structural investigations for a long time and many protein structures of the apo protein and complexes with different ligands have been deposited in the Protein Data Bank (PDB) [44, 45]. Apart from structural methods, MD simulations have been used extensively to study dynamic aspects of the HSA structure [46, 47], as well as to investigate structural and dynamic properties of Cys^34^ [48], which is the predominant thiol in blood and plays a central role as an antioxidant while displaying an unusually low pK_a_ of 8.2 [49, 50].

**Fig. 4:**
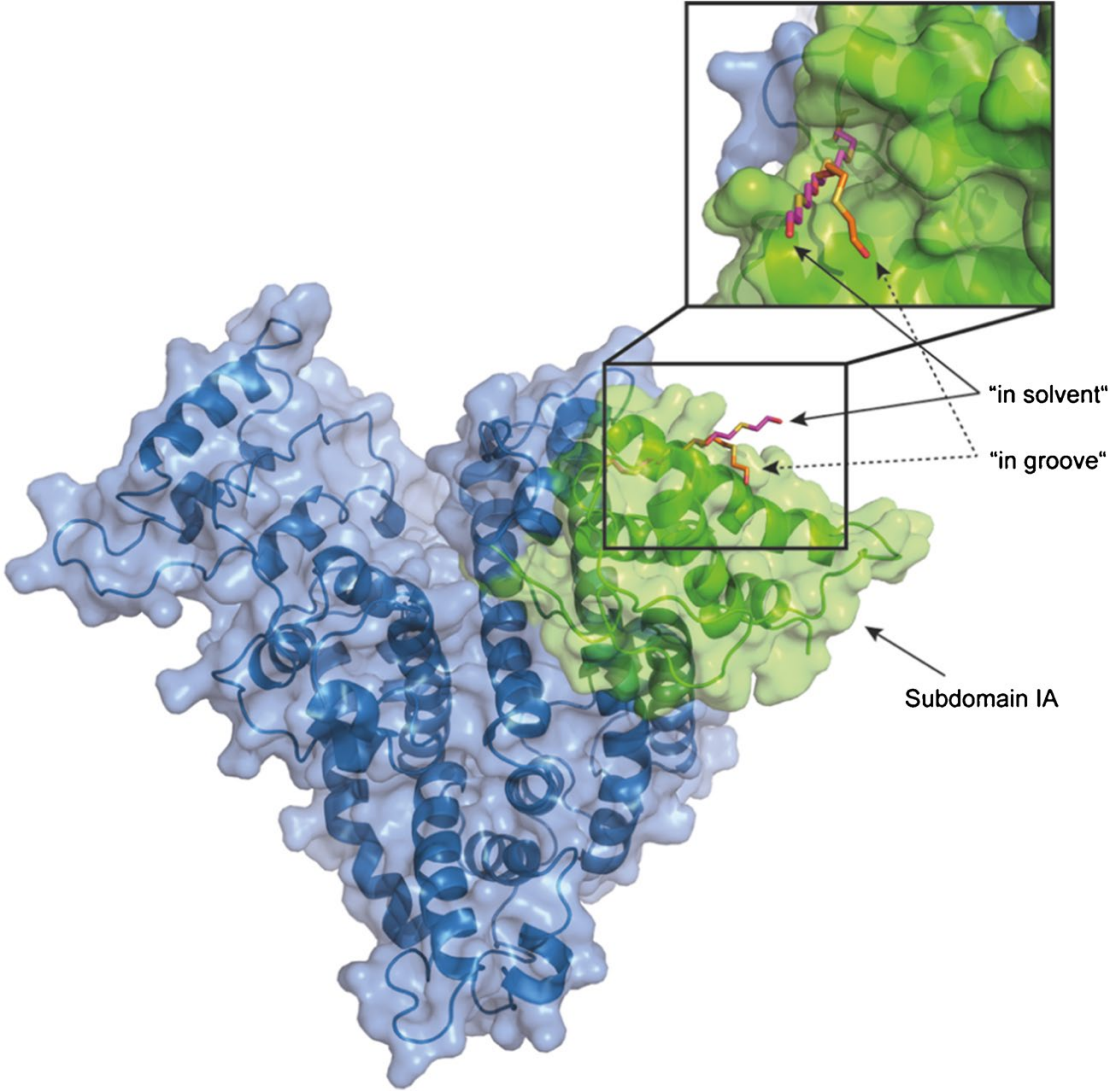
Protein structure of apo HSA (PDB: 1AO6). Subdomain IA is highlighted in green comprising Cys^34^ adducted by T resulting in Cys^34^(-*HETEOETE*). The two starting structures used for MD simulations “in groove” and “in solvent” are shown in stick representation

We used the same simulation frame featuring a solvent-accessible Cys^34^ thiol group from a 200-ns simulation of the apo protein (PDB: 1AO6) to construct the *HETEOETE* adducted variants that we have already used in previous work [14] to create Q-adducted variants of HSA Cys^34^(-*HETETE*). The need to use an MD simulation frame for this purpose is due to the fact that Cys^34^ is only partially solvent accessible and there is sterical hindrance around Cys^34^ in the crystal structure that impedes the construction of adducted variants. As in the case of Cys^34^(-*HETETE*), we constructed two Cys^34^(-*HETEOETE*) variants: the “in solvent” variant in which the *HETEOETE*-moiety points directly into the solvent and the “in groove” variant with the *HETEOETE*-moiety trying to optimize contacts with amino acid side chain in the groove between the two helices adjacent to Cys^34^. Figure 4 shows the spatial orientation of the *HETEOETE*-moiety for both variants. It also highlights the structural domain and subdomain [51] (IA) in which Cys^34^ is located (residues 5–107). A 100-ns MD production run was conducted for each variant. The backbone RMSD of the “in solvent” variant stabilized after about 20–25 ns. For the “in groove” variant, the same was observed after 15–20 ns. For further analysis, only the last 75 ns of each simulation was considered unless otherwise noted. Visual inspection of the two trajectories shows high flexibility of the *HETEOETE*-moiety. This was already observed for the *HETETE*-moiety of Q [14] and is even more pronounced for *HETEOETE* due to the even larger number of rotational degrees of freedom. The hydroxyl group of the *HETEOETE*-moiety regularly explores the solvent but also forms hydrogen bonding interactions with several surface exposed parts of the protein—again a behavior already observed with *HETETE* of Q.

However, the major difference to earlier MD studies with *HETETE* [14] and *HETE* (from SM, unpublished data) is that, regardless of the starting structure (“in solvent” and “in groove”), both simulation trajectories overall showed the same results and converged to a common set of favorable orientations of the *HETETE-* and *HETE*-moiety with respect to the other parts of the protein. In case of *HETEOETE*, this is not the case even though the differences in the starting structures were relatively minor (Fig. 4).

Visual inspection of the two MD trajectories reveals that with the “in groove” starting structure, hydrophobic and polar and hydrogen bonding interactions with Lys^41^, Leu^42^, and Glu^45^ are of high importance and lead to positional arrangements of the *HETEOETE*-moiety somewhat similar to those previously found for *HETETE* [14]. The terminal hydroxyl group forms either hydrogen bonding interactions with the solvent or with the side chains of Glu^45^, Thr^76^, or Glu^37^ (in order of observed frequency).

In case of the “in solvent” starting structure, the trajectory shows intensive interactions with residues of a surface-exposed highly flexible loop (residues 80–89) [52]. After reaching a stable backbone RMSD, the *HETEOETE*-moiety continues to interact with residues further down the loop until a hydrogen bond of the *HETEOETE* hydroxyl group with the side chain as well as the backbone amide of Glu^86^ is observed frequently. This interaction becomes dominant for the remainder of the trajectory.

**Fig. 5:**
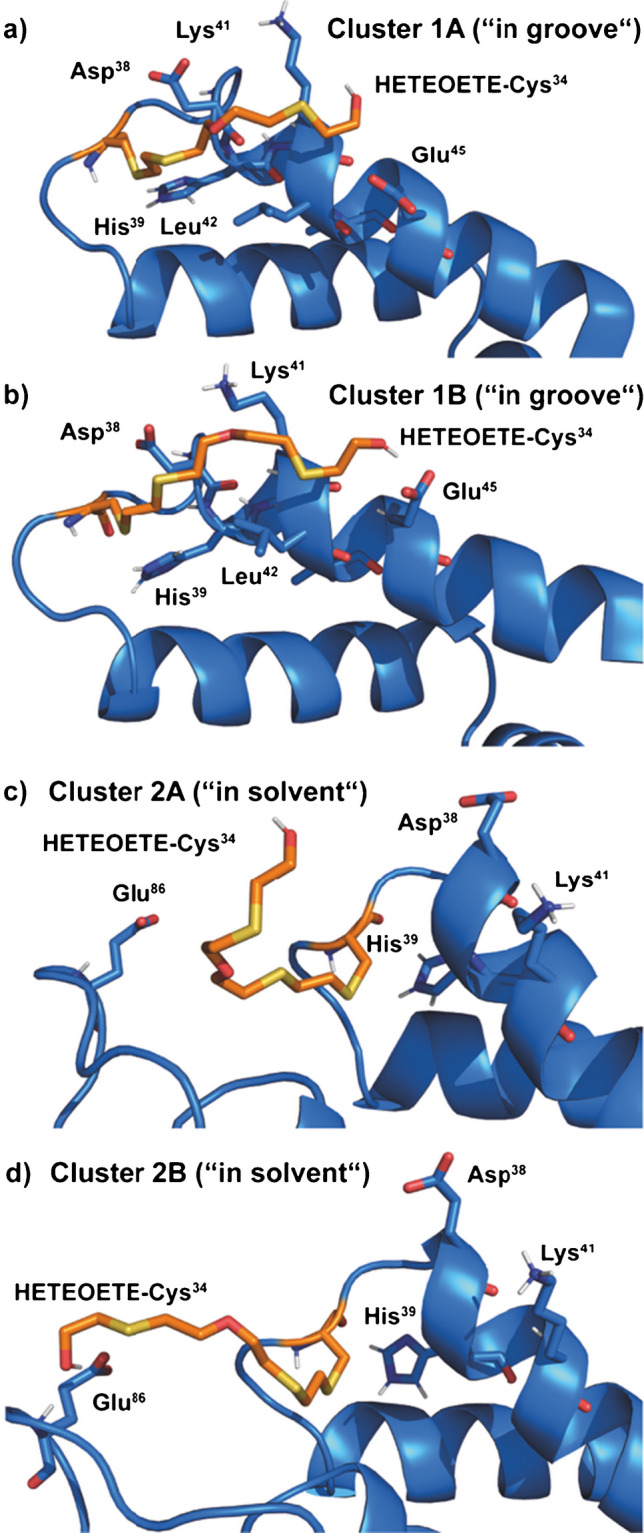
Cluster analysis of MD simulations. Structural environment around *HETEOETE*-Cys^34^ in the two most populated clusters for the “in groove” (1A and 1B) and “in solvent” (2A and 2B) starting structures after cluster analysis. Important hydrogen bonding partners (Glu^46^ and Glu^86^) as well as important hydrophobic interaction partners (Leu^42^ and Lys^41^) are highlighted

Visualization of the different dominating interactions is facilitated by cluster analysis employing the method of Daura et al. [53] as implemented in the GROMACS software package. A total of 7501 simulation frames from the last 75 ns of the trajectory showing a stable backbone RMSD were analyzed by this technique (Fig. 5).

For the “in groove” trajectory, the most populated cluster (76% of frames, Fig. 5a, cluster 1A) shows—in high similarity to the findings with *HETETE* from Q—interactions of the *HETEOETE*-moiety with Glu^45^, Leu^42^, Lys^41^, as well as His^39^ and Asp^38^. Overall, the positioning of the *HETEOETE*-moiety appears more cramped compared to *HETETE* with the hydroxyl group of both moieties located close to Glu^45^ in both cases. The second most populated cluster (10% of frames, Fig. 5b, cluster 1B) shows a similar arrangement compared to cluster 1 but with a hydrogen bond of the *HETEOETE* hydroxyl group and the carboxylate of Glu^45^. The frequency of observation of this hydrogen bond is higher than 10% as it is displayed also in other minor clusters and even some frames of cluster 1 as the displayed structure only show the representative average structure. The real observed value is around 18%.

For the “in solvent” trajectory, the two most populated clusters show a completely different picture. The most populated cluster (38% of frames, Fig. 5c, cluster 2A) shows the *HETEOETE*-moiety mainly in contact with the solvent. The second most populated cluster (32% of frames, Fig. 5d, cluster 2B) shows the hydrogen bonding interaction of *HETEOETE* with Glu^86^. In this structure, the hydroxyl group acts both as a hydrogen bond donor to the Glu^86^ carboxylate and a hydrogen bond acceptor from the backbone amide.

It has previously been shown in vitro that Glu residues in HSA can react with SM [54]. Adducts of SM with Glu in HSA were found at several residues with Glu^230^ being the most prominent one. This finding could also be confirmed in vivo in real cases of human exposure to SM [15]. Even though neither Glu^45^ nor Glu^86^ has been explicitly mentioned in the literature as locations for adduct formation with SM, the MD simulations conducted with *HETEOTE*-modified Cys^34^ clearly show that it is sterically possible for T to first react with Cys^34^ (or Glu^45^/Glu ^86^) and then with the other reactive half of the molecule with one of the two Glu residues (or the Cys^34^ thiol group, respectively). This reaction with both Cys^34^ and one of the two Glu residues would result in the formation of an intramolecular cross-link. We therefore set out to detect the potential existence of such cross-links experimentally.

### Identification of cross-linked peptides

Earlier studies have shown that Cys^34^ is a preferred target for alkylation of HSA by SM, Q, and diverse structural analogues [14–19, 42]. Any cross-links within HSA were never addressed before. In contrast, inter- and intramolecular cross-links have been described to happen in DNA and keratins when exposed to SM [55]. Based on our MD simulations described above, we postulated a linkage from Glu^86^ or Glu^45^ to Cys^34^. After proteolysis of such cross-linked HSA by proteinase K, we expected the presence of a molecule comprising of two *ETEOETE*-linked peptides: one tripeptide containing Cys^34^ (Cys*^34^ProPhe) and the other one containing Glu*^86^ which sequence was hardly predictable. The star marks the amino acid which side chain is adducted for linkage.

**Fig. 6:**
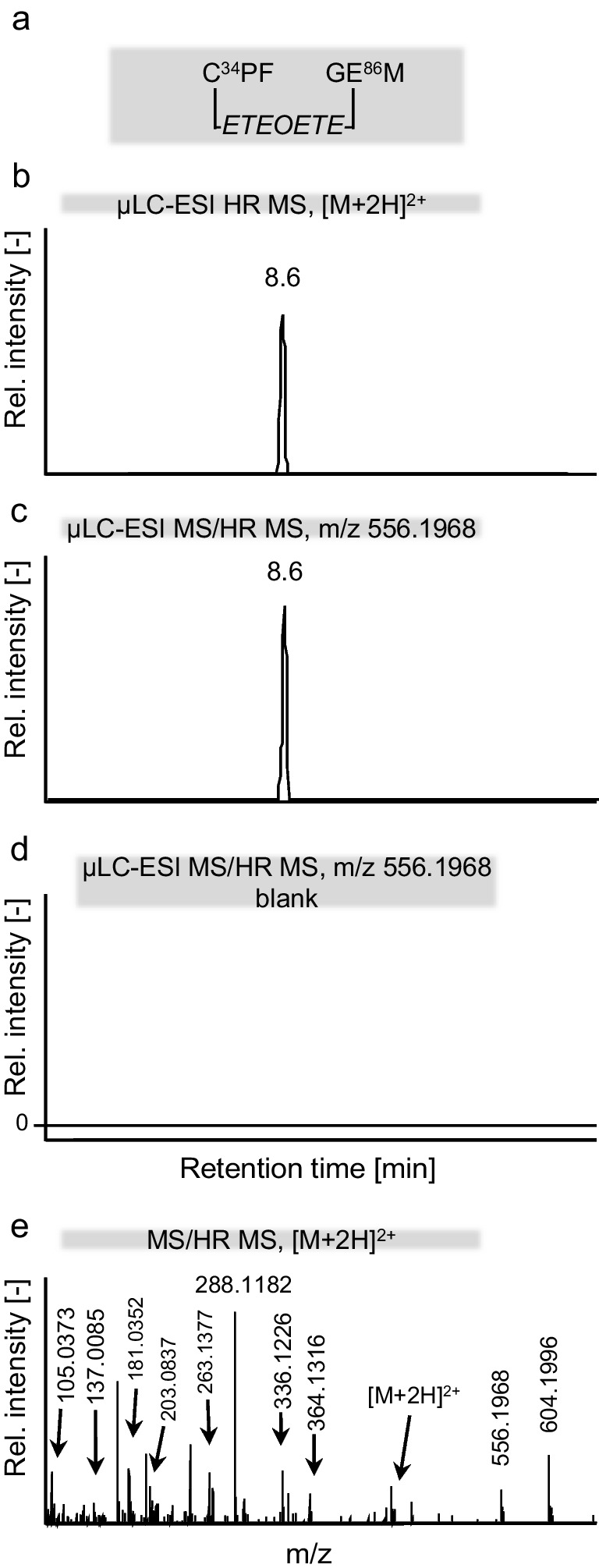
Intramolecular cross-link in HSA by the T-derived *ETEOETE*-linker. **a** Cross-linked peptide obtained from a HSA reference incubated with T after proteolysis with proteinase K. Cys^34^ is linked with Glu^86^ by the ethylthioethyloxyethylthioethyl (*HETEOETE*) linker. This linked peptide Cys^34^*ProPhe(-*ETEOETE*-)GlyGlu^86^*Met was detected by µLC-ESI MS/HR MS (Orbitrap) operating in the data-dependent tandem-MS mode (ddMS2, top 10 [28]) combining full scan MS mode (HR MS) with parallel reaction monitoring (PRM, MS/HR MS). **b** XIC of the double protonated linked peptide ([M + 2H]^2+^ , m/z 446.1596) obtained from HR MS analysis (fwhm 70,000) detected at *t*_R_ 8.6 min. **c** XIC of the product ion at m/z 556.1968 obtained from MS/HR MS analysis (fwhm 17,500) of the precursor ion at m/z 446.1596 (**b**) selectively detecting the linked peptide. This product ion represents the Cys^34^*ProPhe part still bearing the *ETEOETE*-linker (Table ESM 9). **d** XIC of the product ion at m/z 556.1968 obtained from analysis of a plasma blank. The lack of any peak proves the high selectivity of detection and the absence of any interference. **e** MS/HR MS spectrum obtained from the double protonated precursor ion ([M + 2H]^2+^) of the linked peptide (**a**) detected in PRM mode (fwhm 17,500). Structural assignment of labelled product ion signals is given in Table ESM 9 proving the identity of the peptide

The strategy for identification of a postulated cross-linked peptide by µLC-ESI MS/HR MS (Orbitrap) included (i) analysis using the ddMS2 (top 10) approach considering postulated cross-linked peptides in the inclusion list for preferred mass spectrometric fragmentation, (ii) extraction of a chromatographic peak for the mass of the cross-linked peptide from full scan HR MS data, (iii) comparison to corresponding data obtained from a blank to confirm the absence of that peak, and (iv) interpretation of MS/HR MS data to unravel the amino acid sequence and the cross-linker. Characteristic product ions of the linker were detected corresponding to theoretical masses at *m*/*z* 105.0369 ([*HETE*]^+^), *m*/*z* 137.0095 ([*HETET*]^+^), and *m*/*z* 181.0352 ([*HETEOET*]^+^) (Fig. 6e, Table ESM 9).

After proteolysis of T-treated HSA with proteinase K, we succeeded in the detection of the following linked peptides: Cys*^34^ProPhe(-*ETEOETE*-)GlyGlu*^86^Met ([M + 2H]^2+^, *m/z* 446.1596), Cys*^34^ProPhe(-*ETEOETE*-)ThrGlu*^48^ ([M + 2H]^2+^, *m/z* 402.6525), and Cys*^34^ProPhe(-*ETEOETE*-)AsnGlu*^45^ ([M + 2H]^2+^, *m/z* 409.1501). Exemplarily, experimental results for Cys*^34^ProPhe(-*ETEOETE*-)GlyGlu*^86^Met are shown in Fig. 6. The MS/HR MS spectrum of the double protonated linked peptide (Fig. 6e) allowed the unambiguous identification of the linkage. XICs of the peptide (precursor ion at *m*/*z* 446.1596 and a selective product ion at *m*/*z* 556.1968, Table ESM 9) only show one single peak at retention time (*t*_R_) 8.6 min obtained from exposed HSA (Fig. 6b, c) whereas those are lacking in the blank as exemplarily illustrated for the product ion at *m*/*z* 556.1968 (Fig. 6d) thus indicating high selectivity for peptide detection. This linked peptide was also detected when analyzing the much more complex plasma reference thus indicating excellent selectivity of detection. In addition, LeuGlnGlnCys*^34^ProPheGluAsp(-*ETEOETE*-)LeuArgGluThrTyrGlyGlu*^86^Met ([M + 3H]^3+^, *m*/*z* 722.9787) and LeuGlnGlnCys*^34^ProPheGluAsp(-*ETEOETE*-)ValAsnGlu*^45^ValThrGlu ([M + 3H]^3+^, *m*/*z* 620.6029) were found after cleavage with pepsin confirming the cross-linked Cys^34^ residue (data not shown). Furthermore, LeuArgGlu*^82^ThrTyrGlyGluMet, ValAsnGlu*^45^ValThrTrpGlu, ValAsnGluValThrTrpGlu*^48^, and LeuArgGluThrTyrGlyGlu*^86^Met were also detected by HR MS as well as MS/HR MS as alkylated but not cross-linked peptides (data not shown).

Cys*^34^ProPhe(-*ETEOETE*-)GlyGlu*^86^Met was also detected in T-exposed plasma references indicating that despite the presence of numerous non-HSA proteins in plasma, intramolecular cross-links were formed.

## Conclusions

In many aspects, T resembles both SM and Q in the way it forms adducts with HSA. This is for example evident from the formation of adducts with the free thiol group of Cys^34^ of HSA. However, T and also Q are not just long versions of SM but show specific behavior based on their physical and chemical properties. These properties include among others reduced hydrophilicity and increased lipophilicity when compared to SM and a larger number or rotatable bonds in the linker between the two chloroethyl groups resulting in increased conformational flexibility. As a result, we were able to find expected as well as new HSA adducts of T, many of them of high value in the biomedical verification of exposure. The presented biomarkers also extend the number of targets for biomedical verification of exposure to vesicants in general. We present a unified workflow based on µLC-ESI MS/HR MS to detect relevant adducts of SM, Q, and T simultaneously. Q and T are the most prominent synthesis byproducts of SM and are also formed during long-term storage with concentrations varying widely based on synthesis route, precursor quality, and storage duration and conditions. This opens the door to the exploitation of biomedical samples in chemical forensics of sulfur mustards. Analysis of biomedical samples might allow the determination if a characteristic ratio of SM-, Q-, and T-adducts could be caused by a certain batch of sulfur mustard.

For the first time, we have also presented experimental proof for the ability of higher mustards to form intramolecular cross-links in proteins, specifically in HSA. Cross-links may alter structural and physico-chemical as well as physiological properties of proteins, what may be less relevant for HSA but of much more importance for other proteins, e.g., the transient receptor potential ankyrin 1 (TRPA1) channels [56–58]. Cross-links may also appear quite likely in hair keratins exposed to mustard agents causing alkylation of diverse Glu residues as shown recently [18]. This finding might therefore not only be of general scientific interest but also specifically for the molecular mechanism of sulfur mustard toxicity which remains not fully understood even more than 100 years after the first use of SM on the battlefield.

Only recently the OPCW has used Q as a spiking chemical in the 8th Biomedical Proficiency Test for the first time [59]. With the available adducts of Q and its derivatives with varying alkyl linkers and the adducts presented here for T, the biomedical verification toolkit now covers all relevant members of Schedule 1.A.04 of the Chemical Weapons Convention and therefore further increases its deterrent value.

## Supplementary Information

Below is the link to the electronic supplementary material.

Supplementary file1 (PDF 795 KB)

## Abbreviations

ACN: Acetonitrile
Atr-*d*_*3*_: Atropine, triple deuterated
BioPT: OPCW biomedical proficiency test
CE: Collision energy
CID: Collision-induced dissociation
CWC: Chemical Weapons Convention
ESI: Electrospray ionization
FA: Formic acid
fwhm: Full width at half-maximum
*HETE*: Hydroxyethylthioethyl-moiety
*HETETE*: Hydroxyethylthioethylthioethyl-moiety
*HETEOETE*: Hydroxyethylthioethyloxyethylthioethyl-moiety
HR: High-resolution
HSA: Human serum albumin
iPrOH: Isopropanol
LC: Liquid chromatography
µLC: Micro liquid chromatography
LOI: Limit of identification
MD: Molecular dynamics
MS: Mass spectrometry
MS/MS: Tandem-mass spectrometry
NMR: Nuclear magnetic resonance
OPCW: Organisation for the Prohibition of Chemical Weapons
PBS: Phosphate-buffered saline
PIS: Product ion scan
Q: Sesquimustard (1,2-bis(2-chloroethylthio) ethane)
RMSD: Root mean square deviation
RMSF: Root mean square fluctuation
RT: Room temperature
SM: Sulfur mustard (bis(2-chloroethyl) sulfide)
TIC: Total ion chromatogram
t_R_: Retention time
T: Agent T, O-mustard (bis(2-chloroethylthioethyl) ether)
XIC: Extracted ion chromatogram
