# Supplementary material for: The blistering warfare agent O-mustard (agent T) generates protein-adducts with human serum albumin useful for biomedical verification of exposure and forms intramolecular cross-links
Source: Anal Bioanal Chem. 2024 Aug 31;416(26):5791–804. doi: 10.1007/s00216-024-05501-8 (PMC11493803; doi:10.1007/s00216-024-05501-8)

Electronic Supplementary Material

The blistering warfare agent O-mustard (agent T) generates protein-adducts with human serum albumin useful for biomedical verification of exposure and forms intramolecular cross-links

Marc-Michael Blum[1], Wolfgang Schmeißer[2], Marina Dentzel[2], Horst Thiermann[2], Harald John[2*]

[1] Blum – Scientific Services,
Björnsonweg 70d, 22587 Hamburg, Germany

[2] Bundeswehr Institute of Pharmacology and Toxicology,
Neuherbergstraße 11, 80937 Munich, Germany

* Address correspondence to this author.

Bundeswehr Institute of Pharmacology and Toxicology

Neuherbergstrasse 11, 80937 Munich, Germany

Phone: +49-89-992692-2311. Fax: +49-89-992692-2333.

E-mail: HaraldJohn@bundeswehr.org

Table of Content

Table ESM 1: Mass spectrometric parameters and characteristics for detection of biomarker adducts with Q

Table ESM 2: Product ions of single protonated His(-*HETEOETE*)

Table ESM 3: Product ions of single protonated Cys(-*HETEOETE*)ProPhe

Table ESM 4: Product ions of double protonated AlaGlu(-*HETEOETE*)ValSerLysLeu

Table ESM 5: Product ions of single protonated double charged LeuGlyMet(-*HETEOETE*)Phe

Table ESM 6: Product ions of single protonated ValThrGlu(-*HETEOETE*)Phe

Table ESM 7: Product ions of double protonated AlaGlu(-*HETETE*)ValSerLysLeu

Table ESM 8: Product ions of single protonated double charged LeuGlyMet(-*HETETE*)Phe

Table ESM 9: Product ions of double protonated linked peptide Cys^{34*}ProPhe(-*ETEOETE-*)GlyGlu^{86*}Met

Figure ESM 1: HSA-derived biomarkers of exposure to sesquimustard (Q) obtained after pepsin-catalyzed proteolysis

Protocol ESM 1: Adding of Cys(-*HETEOTE*) to the GROMOS96 54a7 force field

Protocol ESM 2: RMSD fluctuation of MD trajectories

Table ESM 1: Mass spectrometric parameters and characteristics for detection of biomarker adducts with Q

Compound	Precursor ion	*m/z*	Qual I [*m/z*] (CE [V])	Qual II [*m/z*] (CE [V])	Qual III [*m/z*] (CE [V])	t_R [min]	Qualifier Ion ratio for LOI
AE^{230}(-*HETETE*)VSKL	$[M+2H]^{2+}$	405.704	646.376 (20)	105.037 (20)	137.009 (25)	5.75	II/I (78.5%)
LGM^{329}(-*HETETE*)F	$[M+H]^{2+}$	316.140	467.232 (10)	105.037 (15)	137.009 (10)	5.38	II/I (53.4%)

CE: collision energy; Qual: qualifier ion; t_R: retention time

The following MS settings were used for simultaneous PIS analysis: ion spray voltage floating (ISVF) 4.5 kV; declustering potential (DP) 60 V; curtain gas (CUR) $2.07 \cdot 10^5$ Pa (30 psi); heater gas (GS1) $2.76 \cdot 10^5$ Pa (40 psi); turbo ion spray gas (GS2) $3.45 \cdot 10^5$ Pa (50 psi); temperature (TEM) 200°C; collision energy spread (CES) 3 V; ion release delay (IRD) 67 ms; ion release width (IRW) 25 ms and accumulation time 50 ms.

Biomarkers were obtained after pepsin-catalyzed proteolysis of HSA-adducts of Q in the plasma reference.

Table ESM 2: Product ions of single protonated His(-*HETEOETE*)

Structure	Formula	Measured mass	Theoretical mass	Δ [ppm]	Δ [mmu]
$[M+H]^+$ 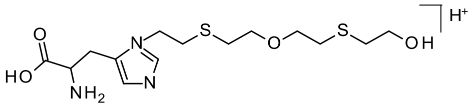	$C_{14}H_{26}N_3O_4S_2$	364.136	364.1359	0.3	0.10
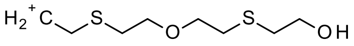	$C_8H_{17}O_2S_2$	209.066	209.0665	-2.4	-0.50
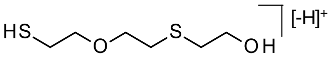	$C_6H_{13}O_2S_2$	181.034	181.0352	-6.6	-1.20
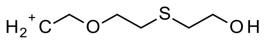	$C_6H_{13}O_2S$	149.063	149.0631	-0.7	-0.10
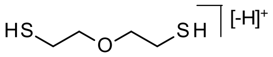	$C_4H_9OS_2$	137.008	137.0089	-6.6	-0.90
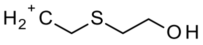	C_4H_9OS	105.036	105.0369	-8.6	-0.90

The corresponding MS/HR MS spectrum is shown in Figure 3b.

Table ESM 3: Product ions of single protonated Cys(-*HETEOETE*)ProPhe

Structure	Formula	Measured mass	Theoretical mass	Δ [ppm]	Δ [mmu]
$[M+H]^+$ 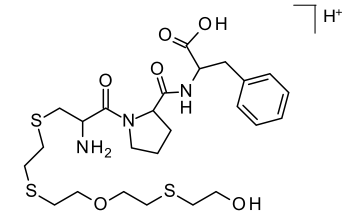	$C_{25}H_{40}N_3O_6S_3$	574.204	574.2074	-5.9	-3.40
$[M-H_2O+H]^+$	$C_{25}H_{38}N_3O_5S_3$	556.204	556.1968	13.0	7.20
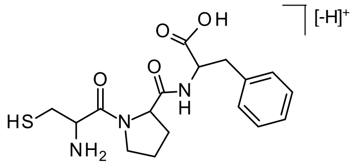	$C_{17}H_{22}O_4N_3S$	364.131	364.1326	-4.4	-1.60
y_2	$C_{14}H_{19}O_3N_2$	263.138	263.1390	-3.8	-1.00
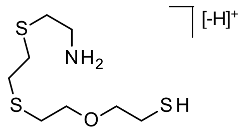	$C_8H_{18}ONS_3$	240.054	240.0545	-2.1	-0.50
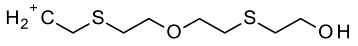	$C_8H_{17}O_2S_2$	209.066	209.0665	-2.4	-0.50
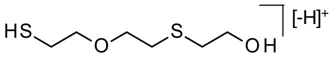	$C_6H_{13}O_2S_2$	181.034	181.0352	-6.6	-1.20
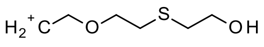	$C_6H_{13}O_2S$	149.063	149.0631	-0.7	-0.10
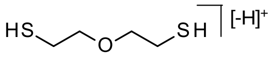	$C_4H_9OS_2$	137.009	137.0089	0.7	0.10
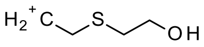	C_4H_9OS	105.037	105.0369	1.0	0.10

The corresponding MS/HR MS spectrum is shown in Figure 3c.

Table ESM 4: Product ions of double protonated AlaGlu(-*HETEOETE*)ValSerLysLeu

Structure	Formula	Measured mass	Theoretical mass	Δ [ppm]	Δ [mmu]
$[M+2H]^{2+}$ AE(-*HETEOETE*)VSKL⌉ $^{2H^{2+}}$	$C_{36}H_{67}N_7O_{12}S_2$	427.720	427.7217 (854.4362 as $[M+H]^+$)	-4.0	-1.70
AE(-*OETE*)VSKL⌉ $^{H^+}$ (loss of $[$*HETE*$]^+$)	$C_{32}H_{60}N_7O_{11}S$	750.407	750.4066	0.5	0.40
AEVSKL: y_6	$C_{28}H_{52}N_7O_{10}$	646.378	646.3770	1.6	1.00
AEVSKL: y_4	$C_{20}H_{40}N_5O_6$	446.299	446.2973	3.8	1.70
AEVSKL: y_3	$C_{15}H_{31}N_4O_6$	347.230	347.2289	3.2	1.10
AEVSKL: y_2	$C_{12}H_{26}N_3O_3$	260.196	260.1969	-3.5	-0.90
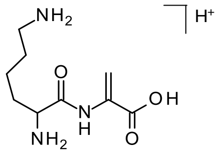	$C_9H_{18}N_3O_3$	216.134	216.1343	-1.4	-0.30
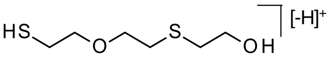	$C_6H_{13}O_2S_2$	181.035	181.0352	-1.1	-0.20
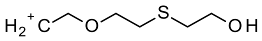	$C_6H_{13}O_2S$	149.063	149.0631	-0.7	-0.10
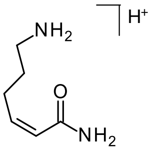	$C_6H_{13}N_2O$	129.102	129.1022	-1.6	-0.20
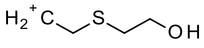	C_4H_9OS	105.037	105.0369	1.0	0.10

AEVSKL denominates the peptide backbone after cleavage of the hydroxyethylthioethyloxyethylthioethyl (*HETEOETE*)-moiety.

The corresponding MS/HR MS spectrum is shown in Figure 3d.

Table ESM 5: Product ions of single protonated double charged LeuGlyMet(-*HETEOETE*)Phe

Structure	Formula	Measured mass	Theoretical mass	Δ [ppm]	Δ [mmu]
$[M+H]^{2+}$<br>LGM(-*HETEOETE*)F ⌉H^{2+}	$C_{30}H_{52}N_4O_7S_3$	338.146	338.1494 (675.2914 as $[M]^+$)	-10.1	-3.40
LGMF ⌉H^+ (loss of [*HETEOETE*]$^+$)	$C_{22}H_{35}O_5SN_4$	467.233	467.2323	1.5	0.70
LGMF: b_4	$C_{22}H_{33}O_4SN_4$	449.221	449.2217	-1.6	-0.70
LGMF: y_3	$C_{16}H_{24}O_4SN_3$	354.150	354.1482	5.1	1.80
LGMF: b_3	$C_{13}H_{24}O_3SN_3$	302.154	302.1533	2.3	0.70
LGMF: y_2	$C_{14}H_{21}O_3SN_2$	297.126	297.1267	-2.4	-0.70
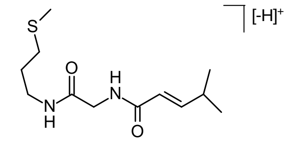	$C_{12}H_{21}O_2SN_2$	257.133	257.1318	4.7	1.20
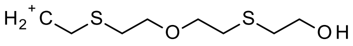	$C_8H_{17}O_2S_2$	209.066	209.0670	-2.4	-0.50
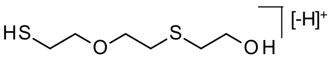	$C_6H_{13}O_2S_2$	181.035	181.0352	-1.1	-0.20
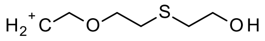	$C_6H_{13}O_2S$	149.063	149.0631	-0.7	-0.10
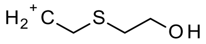	C_4H_9OS	105.036	105.0369	-8.6	-0.90
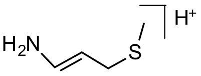	$C_4H_{10}NS$	104.053	104.0529	1.0	0.10

LGMF denominates the peptide backbone after cleavage of the hydroxyethylthioethyloxyethylthioethyl (*HETEOETE*)-moiety.

The corresponding MS/HR MS spectrum is shown in Figure 3e.

Table ESM 6: Product ions of single protonated ValThrGlu(-*HETEOETE*)Phe

Structure	Formula	Measured mass	Theoretical mass	Δ [ppm]	Δ [mmu]
$[M+H]^+$ VTE(-*HETEOETE*)F $]H^+$	$C_{31}H_{51}N_4O_{10}S_2$	703.304	703.3041	-0.1	-0.10
$[M-H_2O+H]^+$	$C_{31}H_{49}N_4O_9S_2$	685.293	685.2936	-0.9	-0.60
$[M-HCOOH+H]^+$	$C_{30}H_{49}N_4O_8S_2$	657.302	657.2986	5.2	3.40
b_3	$C_{22}H_{40}N_3O_8S_2$	538.226	538.2251	1.7	0.90
b_3-H_2O	$C_{22}H_{38}N_3O_7S_2$	520.215	520.2146	0.8	0.40
y_2	$C_{22}H_{35}N_2O_7S_2$	503.188	503.1880	0.0	0.00
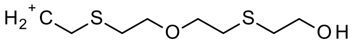	$C_8H_{17}O_2S_2$	209.066	209.0670	-2.4	-0.50
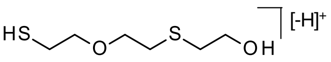	$C_6H_{13}O_2S_2$	181.035	181.0352	-1.1	-0.20
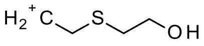	C_4H_9OS	105.036	105.0369	-8.6	-0.90

HETEOETE: hydroxyethylthioethyloxyethylthioethyl-moiety

The corresponding MS/HR MS spectrum is shown in Figure 3f.

Table ESM 7: Product ions of double protonated AlaGlu(-*HETETE*)ValSerLysLeu

Structure	Formula	Measured mass	Theoretical mass	Δ [ppm]	Δ [mmu]
$[M+2H]^{2+}$<br>AE(-*HETETE*)VSKL $\rceil^{2H^{2+}}$	$C_{34}H_{65}N_7O_{11}S_2$	405.704	405.7086<br>(810.4100<br>as $[M+H]^+$)	-11.3	-4.60
AE(-*TE*)VSKL $\rceil^{H^+}$ (loss of $[HETE]^+$)	$C_{30}H_{56}N_7O_{10}S$	706.380	706.3804	-0.6	-0.40
AEVSKL: y_6	$C_{28}H_{52}N_7O_{10}$	646.376	646.3770	-1.6	-1.00
AEVSKL: y_4	$C_{20}H_{40}N_5O_6$	446.296	446.2973	-2.9	-1.30
AEVSKL: b_4	$C_{16}H_{27}N_4O_7$	387.180	387.1784	4.1	1.60
AEVSKL: y_3	$C_{15}H_{31}N_4O_6$	347.229	347.2289	0.3	0.10
AEVSKL: b_3	$C_{13}H_{22}N_3O_5$	300.156	300.1554	2.0	0.60
AEVSKL: y_2	$C_{12}H_{26}N_3O_3$	260.196	260.1969	-3.5	-0.90
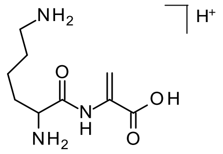	$C_9H_{18}N_3O_3$	216.134	216.1343	-1.4	-0.30
AEVSKL: b_2	$C_8H_{13}N_2O_4$	201.087	201.0870	0.0	0.00
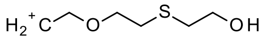	$C_6H_{13}O_2S$	137.009	137.0089	0.7	0.10
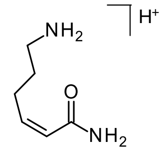	$C_6H_{13}N_2O$	129.102	129.1022	-1.6	-0.20
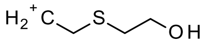	C_4H_9OS	105.036	105.0369	-8.6	-0.90

AEVSKL denominates the peptide backbone after cleavage of the hydroxyethylthioethylthioethyl (*HETETE*)-moiety.

The corresponding MS/HR MS spectrum is shown in Figure ESM 1a.

Table ESM 8: Product ions of single protonated double charged LeuGlyMet(-*HETETE*)Phe

Structure	Formula	Measured mass	Theoretical mass	Δ [ppm]	Δ [mmu]
$[M+H]^{2+}$<br>LGM(-*HETETE*)F $]^{H^{2+}}$	$C_{28}H_{48}N_4O_6S_3$	316.140	316.1363 (631.2652 as $[M]^+$)	11.7	3.70
LGMF $]^{H^+}$ (loss of [*HETETE*]$^+$)	$C_{22}H_{35}O_5SN_4$	467.232	467.2323	-0.6	-0.30
LGMF: b_4	$C_{22}H_{33}O_4SN_4$	449.221	449.2217	-1.6	-0.70
LGMF: y_3	$C_{16}H_{24}O_4SN_3$	354.149	354.1482	2.3	0.80
LGMF: b_3	$C_{13}H_{24}O_3SN_3$	302.154	302.1533	2.3	0.70
LGMF: y_2	$C_{14}H_{21}O_3SN_2$	297.128	297.1267	4.4	1.30
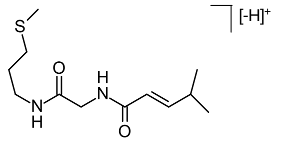	$C_{12}H_{21}O_2SN_2$	257.133	257.1318	4.7	1.20
LGMF: y_1	$C_9H_{12}NO_2$	166.086	166.0863	-1.8	-0.30
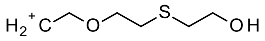	$C_6H_{13}O_2S$	137.008	137.0089	-6.6	-0.90
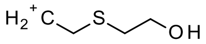	C_4H_9OS	105.036	105.0369	-8.6	-0.90
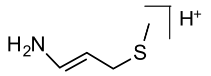	$C_4H_{10}NS$	104.053	104.0529	1.0	0.10

LGMF denominates the peptide backbone after cleavage of the hydroxyethylthioethylthioethyl (*HETETE*)-moiety.

The corresponding MS/HR MS spectrum is shown in Figure ESM 1b.

Table ESM 9: Product ions of double protonated linked peptide

Cys[34*]ProPhe(-*ETEOETE*-)GlyGlu[86*]Met

Structure	Formula	Measured mass	Theoretical mass	Δ [ppm]	Δ [mmu]
$[M+2H]^{2+}$ 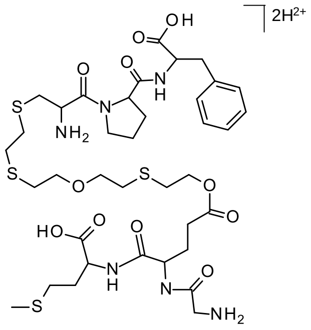	$C_{37}H_{60}N_6O_{11}S_4$	446.1601	446.1596 (891.3119 as $[M+H]^+$)	1.1	0.50
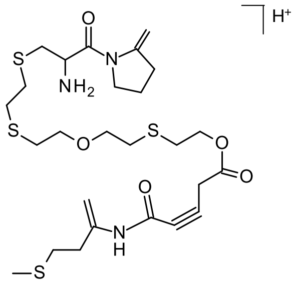	$C_{26}H_{42}N_3O_5S_4$	604.1996	604.2002	-1.0	-0.60
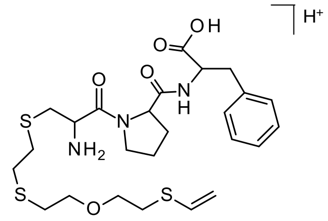	$C_{25}H_{38}N_3O_5S_3$ (single charged ion)<br>$C_{25}H_{39}N_3O_5S_3$ (double charged ion)	556.1968<br>278.6027	556.1968<br>278.6020	0.0<br>2.4	0.00<br>0.70
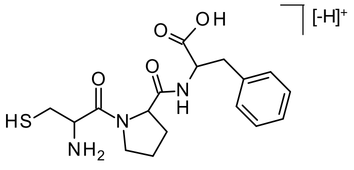	$C_{17}H_{22}N_3O_4S$	364.1316	364.1326	-2.6	-1.00
y_3 (GEM)	$C_{12}H_{22}N_3O_6S$	336.1226	336.1224	0.7	0.20
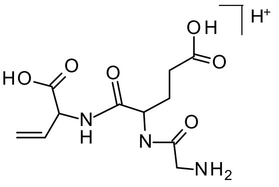	$C_{11}H_{18}N_3O_6$	288.1182	288.1190	-2.8	-0.80
y_2 (PF)	$C_{14}H_{19}N_2O_3$	263.1377	263.1390	-5.0	-1.30

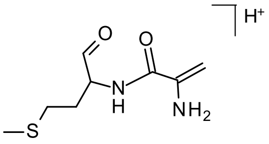	$C_8H_{15}N_2O_2S$	203.0837	203.0849	-5.8	-1.20
b_2 (GE)	$C_7H_{11}N_2O_4$	187.0704	187.0713	-5.0	-0.90
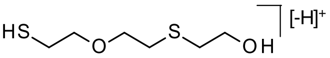	$C_6H_{13}O_2S_2$	181.0352	181.0352	0.3	0.10
y_1 (F)	$C_9H_{12}NO_2$	166.0859	166.0863	-2.2	-0.40
y_1 (M)	$C_5H_{12}NO_2S$	150.058	150.0583	-2.2	-0.30
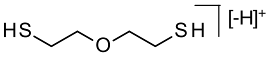	$C_4H_9OS_2$	137.0085	137.0089	-3.1	-0.40
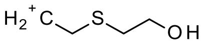	C_4H_9OS	105.0373	105.0369	4.2	0.40

Table ESM 9: Product ions of double protonated linked peptide

Cys^{34*}ProPhe(-*ETEOETE*-)GlyGlu^{86*}Met (continued)

The corresponding MS/HR MS spectrum is shown in Figure 6e.

Figure ESM 1

HSA-derived biomarkers of exposure to sesquimustard (Q) obtained after pepsin-catalyzed proteolysis

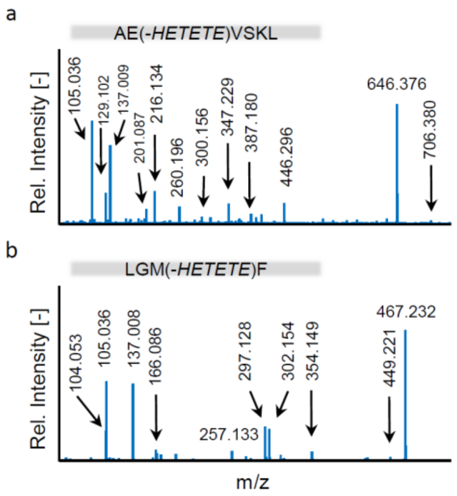

Spectra were extracted from µLC-ESI MS/HR MS (TT5600$^+$) analysis in the product ion scan (PIS) mode of a plasma reference incubated with Q (60 µM). Precursor ions were at (a) *m/z* 405.1 for AE(-*HETETE*)VSKL (Table ESM 7) and (b) *m/z* 316.1 for LGM(-*HETETE*)F (Table ESM 8). Tables referred to in parentheses provide the structural assignment of product ion signals labelled in the spectra.

Protocol ESM 1: Adding of Cys(-*HETEOTE*) to the GROMOS96 54a7 force field

Output ITP file of Automated Topology Builder (ATB) v.3.0 for Cys-HETEOTE (Molecule ID 705026)

```
[ moleculetype ]
; Name   nrexcl
WXVP     3
[ atoms ]
;  nr  type  resnr  resid  atom  cgnr  charge    mass
    1  HS14    1    WXVP    H23    1    0.307   1.0080
    2    OA    1    WXVP     O4    2   -0.475  15.9994
    3  CPos    1    WXVP    C11    3    0.497  12.0110
    4 OEOpt    1    WXVP     O3    4   -0.564  15.9994
    5  CPos    1    WXVP     C1    5    0.510  12.0110
    6    HC    1    WXVP     H3    6   -0.104   1.0080
    7  NPri    1    WXVP     N1    7   -0.836  14.0067
    8  HS14    1    WXVP     H1    8    0.340   1.0080
    9  HS14    1    WXVP     H2    9    0.340   1.0080
   10   CH2    1    WXVP     C2   10    0.171  14.0270
   11     S    1    WXVP     S1   11   -0.382  32.0600
   12   CH2    1    WXVP     C3   12    0.199  14.0270
   13   CH2    1    WXVP     C4   13    0.229  14.0270
   14     S    1    WXVP     S2   14   -0.493  32.0600
   15   CH2    1    WXVP     C5   15    0.251  14.0270
   16   CH2    1    WXVP     C6   16    0.282  14.0270
   17    OE    1    WXVP     O1   17   -0.546  15.9994
   18   CH2    1    WXVP     C7   18    0.262  14.0270
   19   CH2    1    WXVP     C8   19    0.270  14.0270
   20     S    1    WXVP     S3   20   -0.463  32.0600
   21   CH2    1    WXVP     C9   21    0.172  14.0270
   22   CH2    1    WXVP    C10   22    0.304  14.0270
   23  OAlc    1    WXVP     O2   23   -0.640  15.9994
   24  HS14    1    WXVP    H22   24    0.369   1.0080
; total charge of the molecule:   0.000
[ bonds ]
;  ai   aj  funct   c0         c1
    1    2    2   0.1000   1.5700e+07
    2    3    2   0.1340   1.0500e+07
    3    4    2   0.1220   2.2843e+07
    3    5    2   0.1540   4.2166e+06
```

```
    5    6    2   0.1090   1.2300e+07
    5    7    2   0.1470   8.7100e+06
    5   10    2   0.1540   4.2166e+06
    7    8    2   0.1020   1.7782e+07
    7    9    2   0.1020   1.7782e+07
   10   11    2   0.1830   5.6200e+06
   11   12    2   0.1840   6.9412e+05
   12   13    2   0.1530   7.1500e+06
   13   14    2   0.1830   5.6200e+06
   14   15    2   0.1840   6.9412e+05
   15   16    2   0.1530   7.1500e+06
   16   17    2   0.1430   8.1800e+06
   17   18    2   0.1430   8.1800e+06
   18   19    2   0.1530   7.1500e+06
   19   20    2   0.1830   5.6200e+06
   20   21    2   0.1850   1.0665e+06
   21   22    2   0.1530   7.1500e+06
   22   23    2   0.1430   8.1800e+06
   23   24    2   0.0972   1.9581e+07
[ pairs ]
;  ai   aj  funct  ;  all 1-4 pairs but the ones excluded in GROMOS itp
    1    4    1
    1    5    1
    2    6    1
    2    7    1
    2   10    1
    3    8    1
    3    9    1
    3   11    1
    4    6    1
    4    7    1
    4   10    1
    5   12    1
    6    8    1
    6    9    1
    6   11    1
    7   11    1
    8   10    1
    9   10    1
   10   13    1
   11   14    1
   12   15    1
```

```
   13   16    1
   14   17    1
   15   18    1
   16   19    1
   17   20    1
   18   21    1
   19   22    1
   20   23    1
   21   24    1
[ angles ]
;  ai   aj   ak  funct   angle     fc
    1    2    3    2    104.00   490.00
    2    3    4    2    124.00   730.00
    2    3    5    2    115.00   610.00
    4    3    5    2    126.00   640.00
    3    5    6    2    106.75   503.00
    3    5    7    2    108.00   465.00
    3    5   10    2    109.50   520.00
    6    5    7    2    113.00   545.00
    6    5   10    2    109.00  1680.51
    7    5   10    2    111.00   530.00
    5    7    8    2    109.50   425.00
    5    7    9    2    109.50   425.00
    8    7    9    2    107.00  2726.16
    5   10   11    2    113.00   545.00
   10   11   12    2    100.00   475.00
   11   12   13    2    113.00   545.00
   12   13   14    2    113.00   545.00
   13   14   15    2    100.00   475.00
   14   15   16    2    113.00   545.00
   15   16   17    2    109.50   520.00
   16   17   18    2    109.50   450.00
   17   18   19    2    111.00   530.00
   18   19   20    2    113.00   545.00
   19   20   21    2    100.00   475.00
   20   21   22    2    113.00   545.00
   21   22   23    2    111.00   530.00
   22   23   24    2    109.50   450.00
[ dihedrals ]
; GROMOS improper dihedrals
;  ai   aj   ak   al  funct   angle     fc
    3    2    4    5    2      0.00   167.36
```

```
    5    7   10    3    2     35.26   334.72
[ dihedrals ]
;  ai   aj   ak   al  funct    ph0      cp     mult
    1    2    3    5    1    180.00    16.70    2
    4    3    5    7    1    180.00     1.00    6
    5   10   11   12    1      0.00     2.93    3
    7    5   10   11    1      0.00     5.92    3
   10    5    7    8    1      0.00     3.77    6
   10   11   12   13    1    180.00     1.00    3
   11   12   13   14    1      0.00     5.92    3
   12   13   14   15    1      0.00     2.93    3
   13   14   15   16    1      0.00     2.93    3
   14   15   16   17    1      0.00     5.92    3
   15   16   17   18    1      0.00     1.26    3
   16   17   18   19    1      0.00     1.26    3
   17   18   19   20    1      0.00     5.92    3
   18   19   20   21    1      0.00     2.93    3
   19   20   21   22    1    180.00     1.00    3
   20   21   22   23    1      0.00     5.92    3
   21   22   23   24    1      0.00     1.26    3
[ exclusions ]
;  ai   aj  funct  ;  GROMOS 1-4 exclusions
```

Based on this topology output a new entry was generated in the file aminoacids.rtp in the folder of the forcefield for residue CYSX (using Methionine as a starting template)

```
[ CYSX ]
 [ atoms ]
      N     N    -0.31000     0
      H     H     0.31000     0
     CA   CH1     0.00000     1
     CB   CH2     0.24100     2
     SG     S    -0.48200     2
     CD   CH2     0.24100     2
     CE   CH2     0.24100     3
     S2     S    -0.48200     3
     C3   CH2     0.24400     3
     C4   CH2     0.27000     4
     O1    OE    -0.54600     4
     C5   CH2     0.27000     4
     C6   CH2     0.24400     5
     S3     S    -0.48200     5
```

```
      C7   CH2     0.24100     5
      C8   CH2     0.24100     6
      O9    OA    -0.64900     6
     H10     H     0.40800     6
       C     C       0.450     7
       O     O      -0.450     7
[ bonds ]
       N     H    gb_2
       N    CA    gb_21
      CA    CB    gb_27
      CA     C    gb_27
      CB    SG    gb_32
      SG    CD    gb_32
      CD    CE    gb_27
      CE    S2    gb_32
      S2    C3    gb_32
      C3    C4    gb_27
      C4    O1    gb_18
      O1    C5    gb_18
      C5    C6    gb_32
      C6    S3    gb_32
      S3    C7    gb_32
      C7    C8    gb_27
      C8    O9    gb_18
      O9   H10    gb_1
       C     O    gb_5
       C    +N    gb_10
[ angles ]
   ;  ai    aj    ak   gromos type
      -C     N     H     ga_32
      -C     N    CA     ga_31
       H     N    CA     ga_18
       N    CA    CB     ga_13
       N    CA     C     ga_13
      CB    CA     C     ga_13
      CA    CB    SG     ga_16
      CB    SG    CD     ga_4
      SG    CD    CE     ga_16
      CD    CE    S2     ga_16
      CE    S2    C3     ga_4
      S2    C3    C4     ga_16
      C3    C4    O1     ga_13
```

```
      C4    O1    C5     ga_12
      O1    C5    C6     ga_13
      C5    C6    S3     ga_16
      C6    S3    C7     ga_4
      S3    C7    C8     ga_16
      C7    C8    O9     ga_13
      C8    O9    H10    ga_12
      CA     C     O     ga_30
      CA     C    +N     ga_19
       O     C    +N     ga_33
[ impropers ]
   ;  ai    aj    ak    al   gromos type
       N    -C    CA     H     gi_1
      CA     N     C    CB     gi_2
       C    CA    +N     O     gi_1
[ dihedrals ]
   ;  ai    aj    ak    al   gromos type
     -CA    -C     N    CA     gd_14
      -C     N    CA     C     gd_44
      -C     N    CA     C     gd_43
       N    CA    CB    SG     gd_34
       N    CA     C    +N     gd_45
       N    CA     C    +N     gd_42
      CA    CB    SG    CD     gd_26
      CB    SG    CD    CE     gd_26
      SG    CD    CE    S2     gd_34
      CD    CE    S2    C3     gd_26
      CE    S2    C3    C4     gd_26
      S2    C3    C4    O1     gd_34
      C3    C4    O1    C5     gd_23
      C4    O1    C5    C6     gd_23
      O1    C5    C6    S3     gd_34
      C5    C6    S3    C7     gd_26
      C6    S3    C7    C8     gd_26
      S3    C7    C8    O9     gd_34
      C7    C8    O9    H10    gd_23
```

The following entry for CYSX was added to the file aminoacids.hdb to account for hydrogen bonding.

```
CYSX    2
1       1       H       N       -C      CA
1       2       H10     O9      C8      C7
```

After constructing the HETEOETE-moiety using UCSF Chimera the modified residue was named CYSX in the PDB file and the atom names were assigned based on the entry under [atoms] in the aminoacids.rtp file:

CA—CB—SG—CD—CE—S2—C3—C4—O1—C5—C6—S3—C7—C8—O9—H10

Protocol ESM 2: RMSD fluctuation of MD trajectories

HSA apo (200ns)
(from Blum MM, Richter A, Siegert M, Thiermann H, John H. Adduct of the blistering warfare agent sesquimustard with human serum albumin and its mass spectrometric identification for biomedical verification of exposure. Anal Bioanal Chem. 2020; 412:7732–7737.)

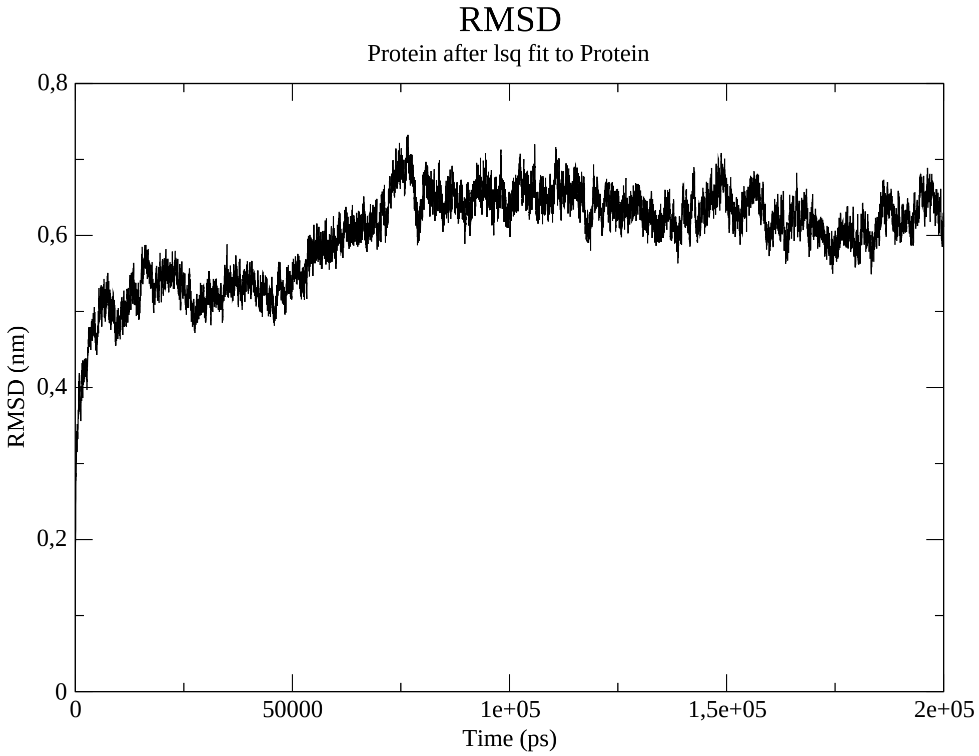

HSA-HETEOETE (100ns) “in solvent”

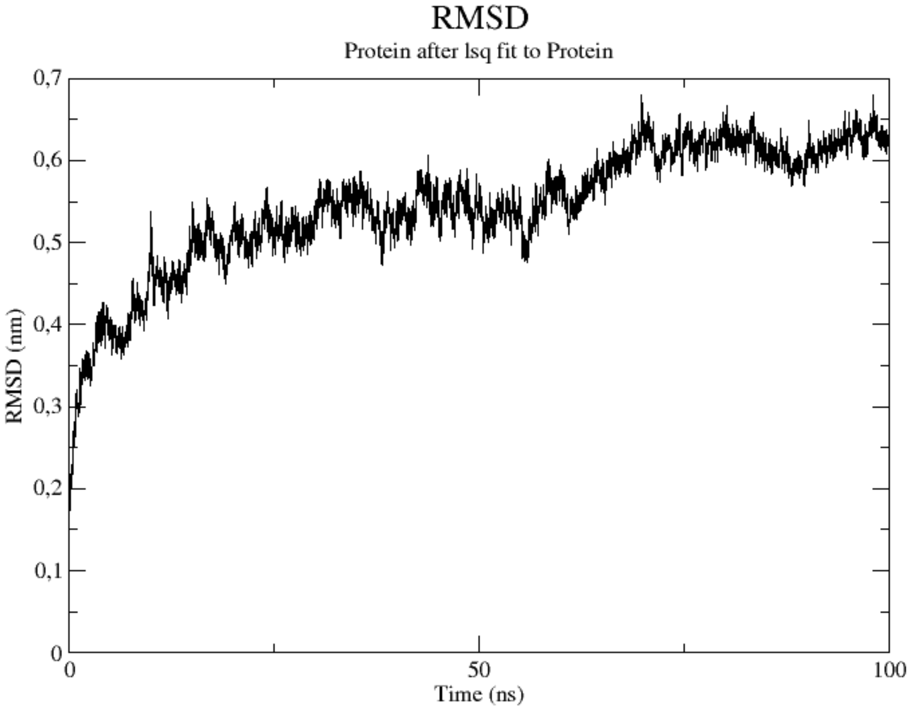

HSA-HETEOETE (100ns) “in groove”

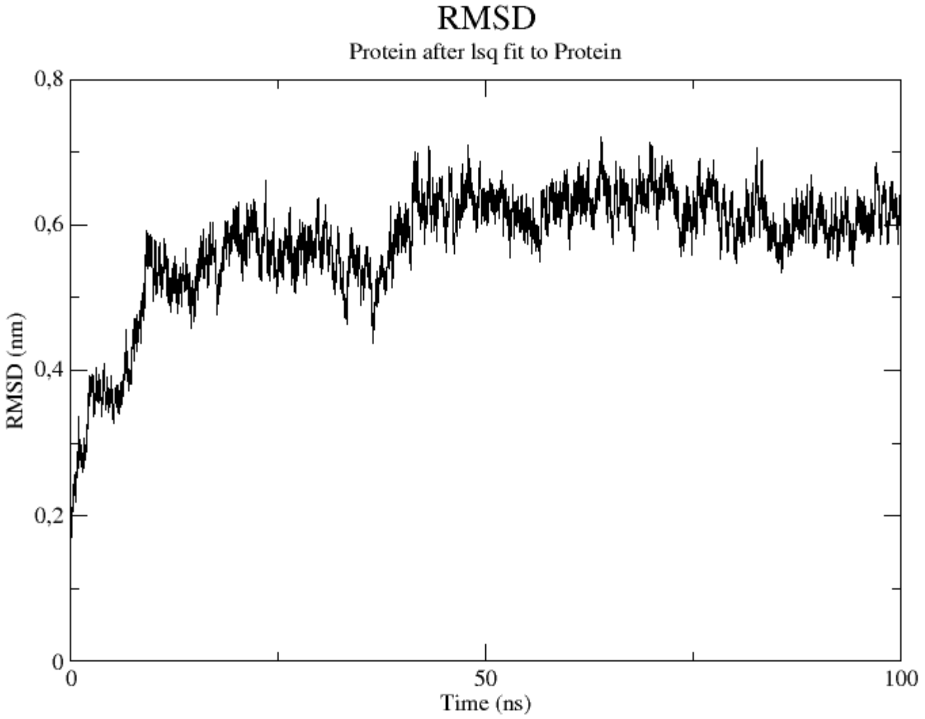

1. RMSF of residues over the course of the MD simulation with converged RMSD

(C-α group used for RMS calculation, light grey section depicting subdomain IA)

HSA apo (100ns – 200ns)

(from Blum MM, Richter A, Siegert M, Thiermann H, John H. Adduct of the blistering warfare agent sesquimustard with human serum albumin and its mass spectrometric identification for biomedical verification of exposure. Anal Bioanal Chem. 2020; 412:7732–7737.)

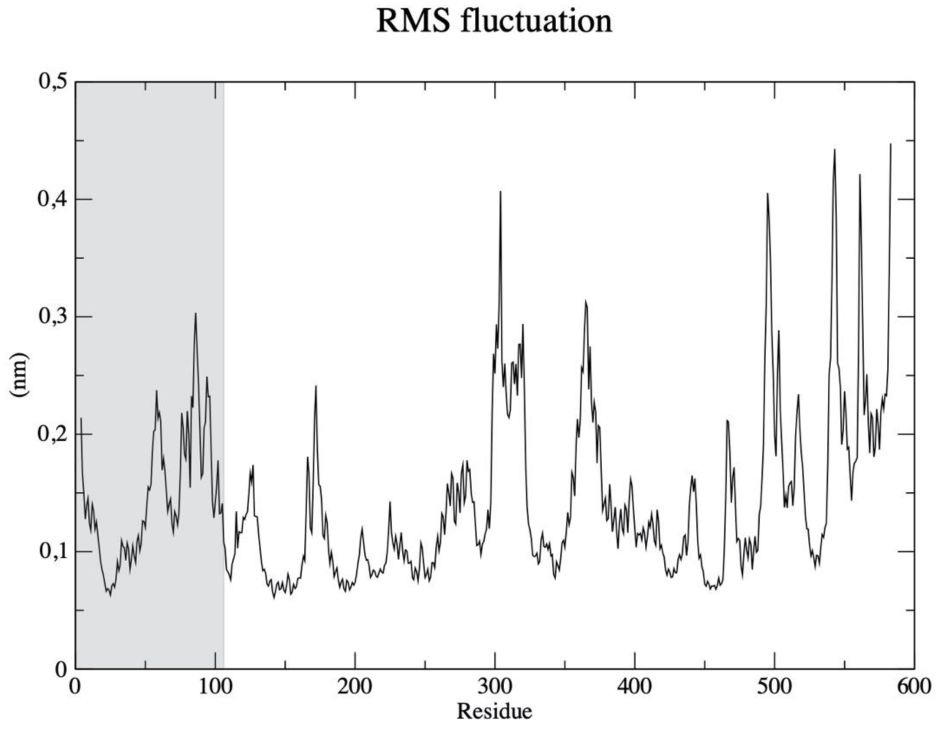

HSA-HETEOETE "in solvent" (60ns – 100ns)

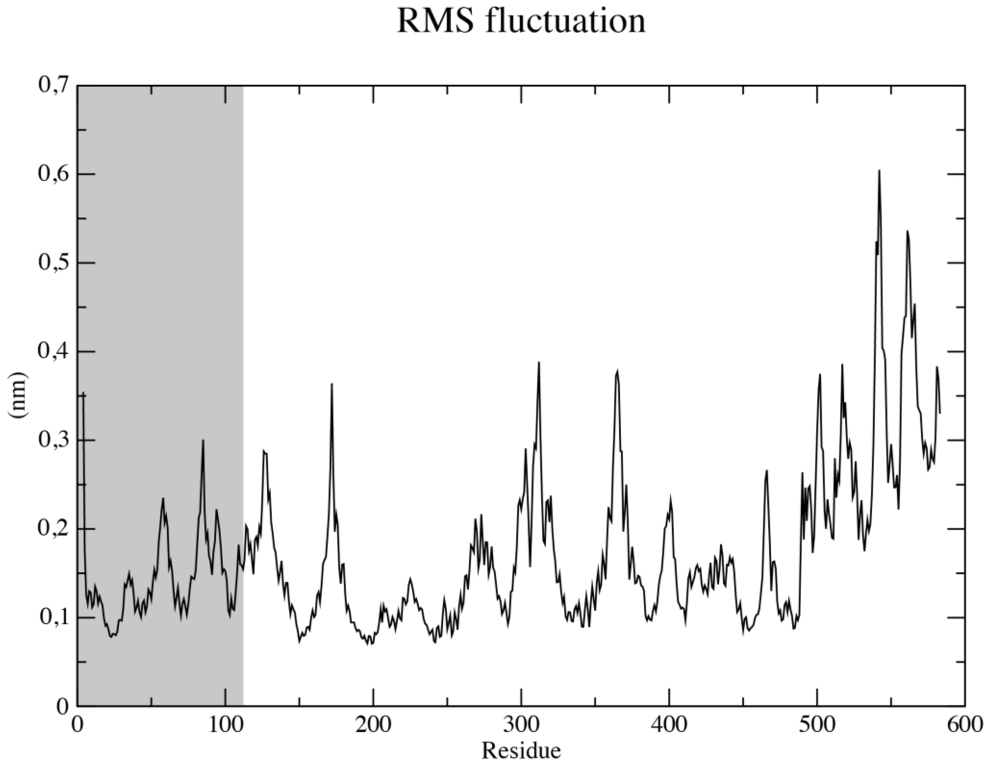

HSA-HETEOETE "in groove" (35ns – 100ns)

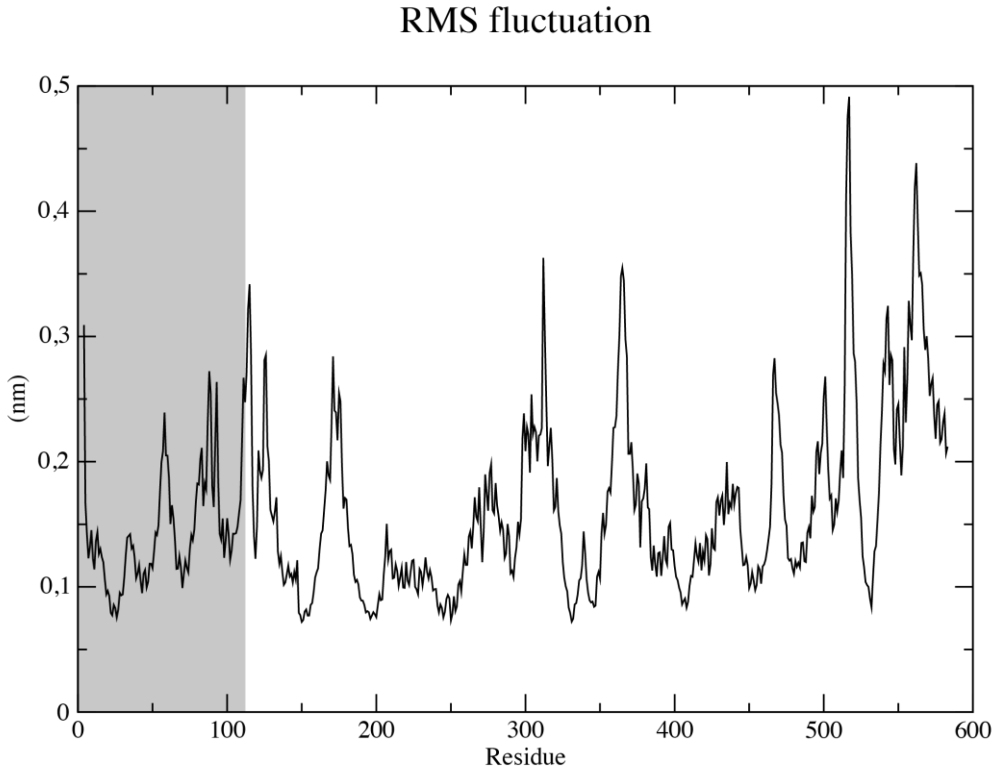

Supplement: Supplementary file 1 — Supplementary file1 (PDF 795 KB) [file 216_2024_5501_MOESM1_ESM.pdf]
